# Supplementary figures and images for: Misfolded proteins bind and activate death receptor 5 to trigger apoptosis during unresolved endoplasmic reticulum stress
Source: eLife. 2020 Jan 6;9:e52291. doi: 10.7554/eLife.52291 (PMC7041945; doi:10.7554/eLife.52291)

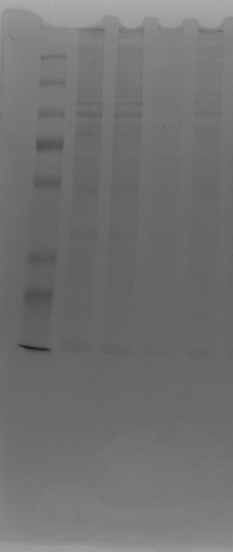

Supplement: Figure 1—source data 1. — This zip archive contains the measured luminescent units for caspase glo 8 activity shown in Figure 1G (IP beads) and Figure 1—figure supplement 3C (input lysates). Coomassie gels used to normalize lysate concentration are included as. tif files. [file elife-52291-fig1-data1.zip › Figure 1 - Source Data 1/Source Data 1S3 inputs/Source Data Fig 1S3 - Coomassie Quant Rep 3.tif]

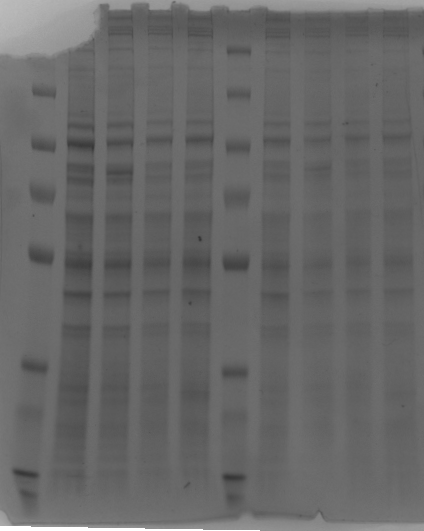

Supplement: Figure 1—source data 1. — This zip archive contains the measured luminescent units for caspase glo 8 activity shown in Figure 1G (IP beads) and Figure 1—figure supplement 3C (input lysates). Coomassie gels used to normalize lysate concentration are included as. tif files. [file elife-52291-fig1-data1.zip › Figure 1 - Source Data 1/Source Data 1S3 inputs/Source Data Fig 1S3 - Coomassie Quant Rep 1 and 2.tif]

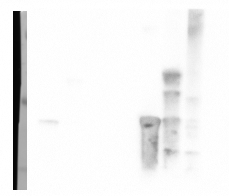

Supplement: Figure 1—source data 2. — This zip archive contains images of the Western blots and measurements used to quantify the amount of DR5 in the IP samples relative to the input lysate. [file elife-52291-fig1-data2.zip › Figure 1 - Source Data 2/Westerns for INS and RHO IP/Rep 2/2018-04-17 GFPTrapIP HCT INS RHO anti-GFP_Exposure_30.0sec-1.tif]

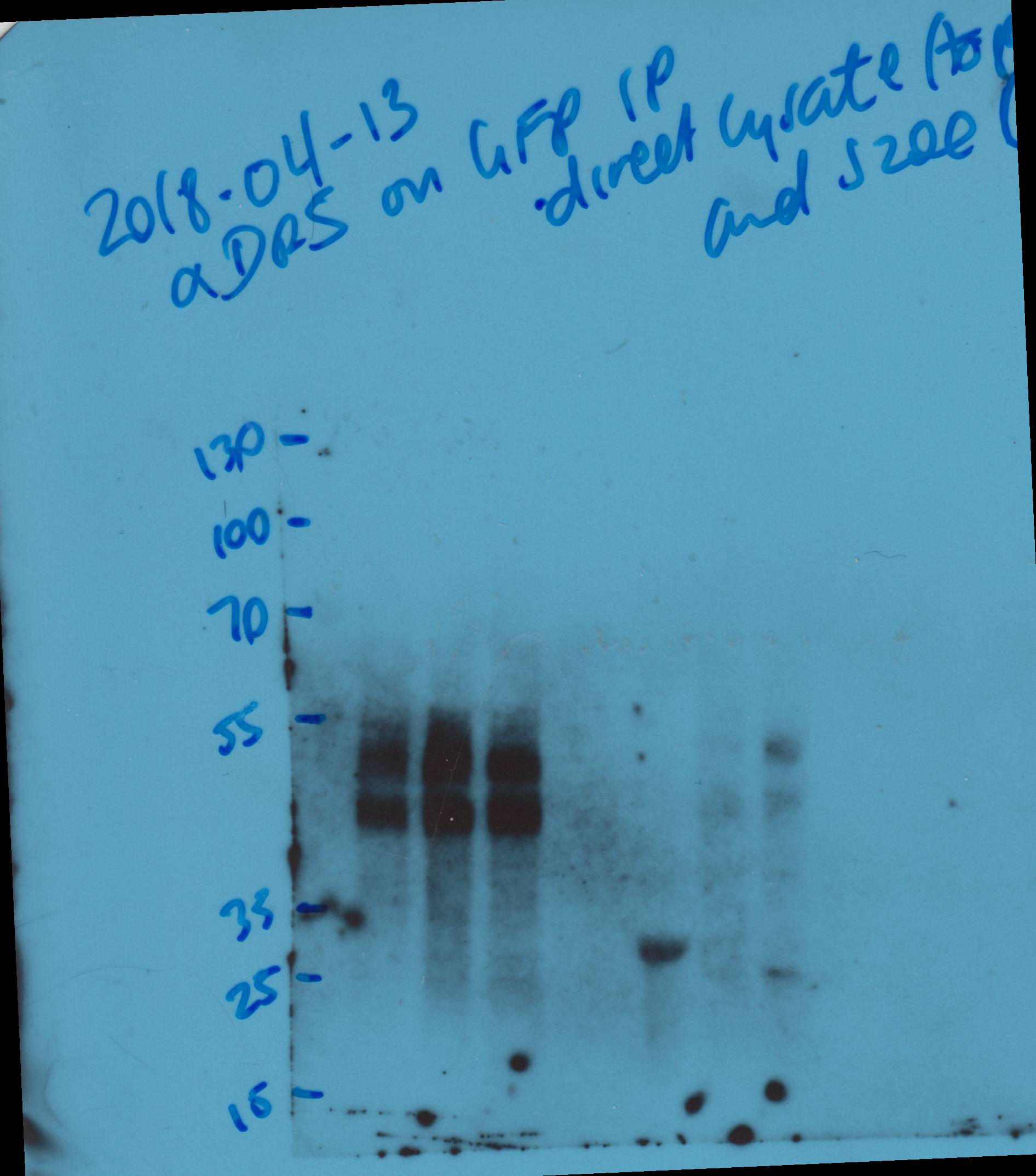

Supplement: Figure 1—source data 2. — This zip archive contains images of the Western blots and measurements used to quantify the amount of DR5 in the IP samples relative to the input lysate. [file elife-52291-fig1-data2.zip › Figure 1 - Source Data 2/Westerns for INS and RHO IP/Rep 2/2018-04-13 anti-DR5 INS1 RHO traffo GFP IP-longexp.tif]

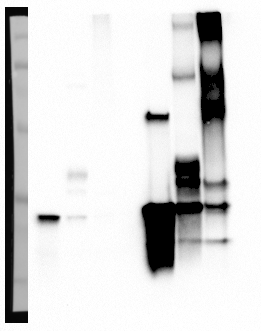

Supplement: Figure 1—source data 2. — This zip archive contains images of the Western blots and measurements used to quantify the amount of DR5 in the IP samples relative to the input lysate. [file elife-52291-fig1-data2.zip › Figure 1 - Source Data 2/Westerns for INS and RHO IP/Rep 1/2018-09-28 anti-GFP GFPTrap IP HCT116 INS RHO_Exposure_20.0sec-short.tif]

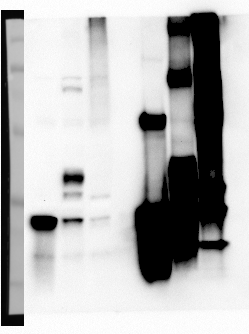

Supplement: Figure 1—source data 2. — This zip archive contains images of the Western blots and measurements used to quantify the amount of DR5 in the IP samples relative to the input lysate. [file elife-52291-fig1-data2.zip › Figure 1 - Source Data 2/Westerns for INS and RHO IP/Rep 1/2018-09-28 anti-GFP GFPTrap IP HCT116 INS RHO_Exposure_160.0sec-long.tif]

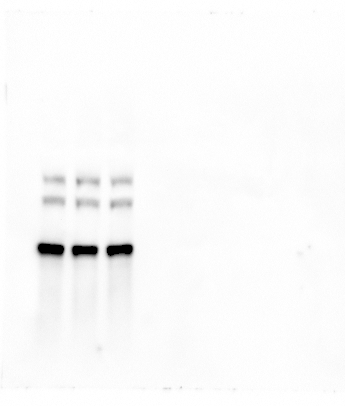

Supplement: Figure 1—source data 2. — This zip archive contains images of the Western blots and measurements used to quantify the amount of DR5 in the IP samples relative to the input lysate. [file elife-52291-fig1-data2.zip › Figure 1 - Source Data 2/Westerns for INS and RHO IP/Rep 1/2018-09-24 anti-DR5 HCT116 INS RHO GFP IP inputs_Exposure_60.0sec.tif]

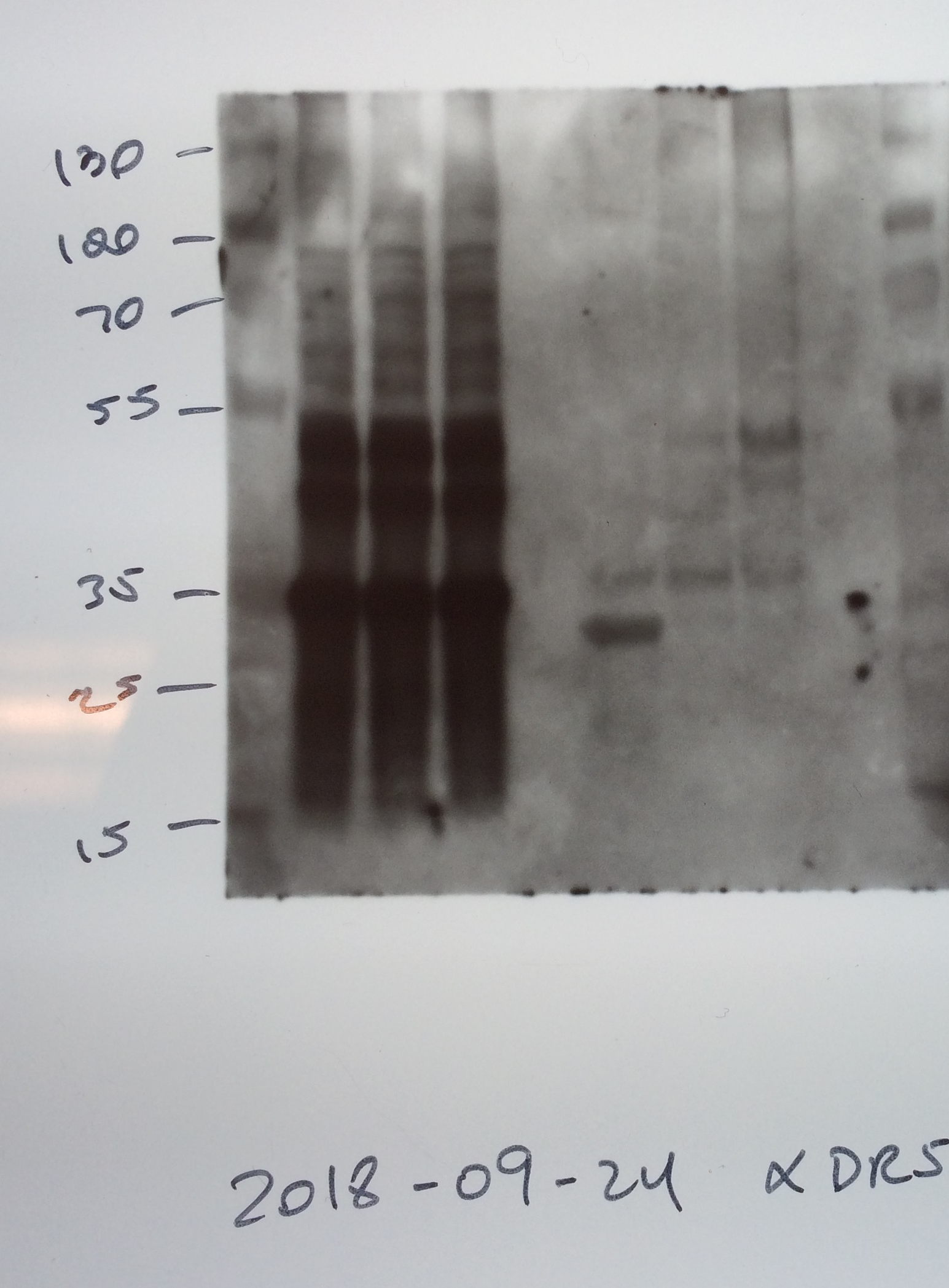

Supplement: Figure 1—source data 2. — This zip archive contains images of the Western blots and measurements used to quantify the amount of DR5 in the IP samples relative to the input lysate. [file elife-52291-fig1-data2.zip › Figure 1 - Source Data 2/Westerns for INS and RHO IP/Rep 1/2018-09-24 anti-DR5 INS1 RHO traffo GFP IP-film.tif]

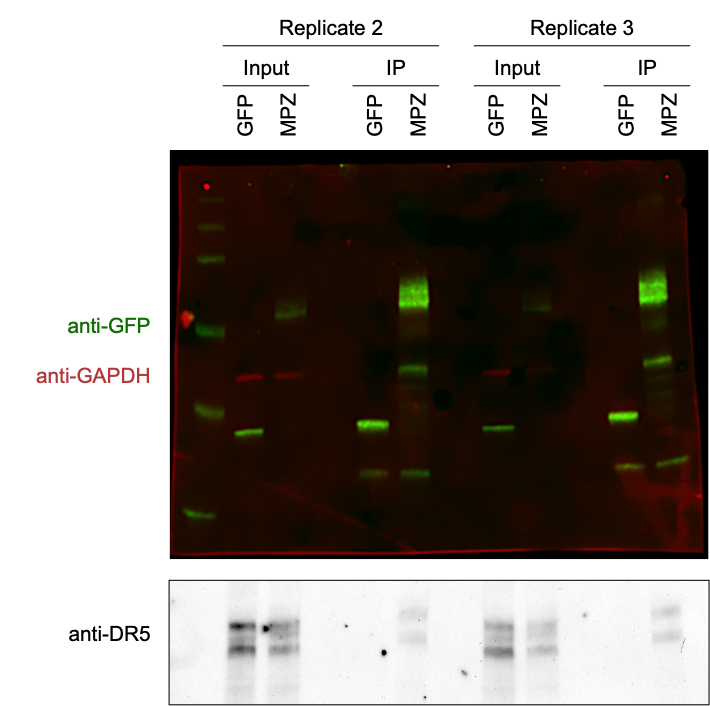

Supplement: Figure 1—source data 2. — This zip archive contains images of the Western blots and measurements used to quantify the amount of DR5 in the IP samples relative to the input lysate. [file elife-52291-fig1-data2.zip › Figure 1 - Source Data 2/Westerns for MPZ IP/Rep 2 and 3/Legend - Rep 2 and 3.png]

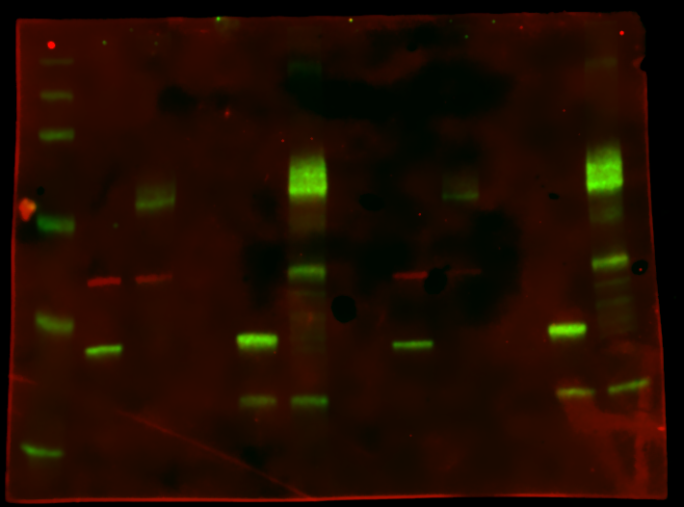

Supplement: Figure 1—source data 2. — This zip archive contains images of the Western blots and measurements used to quantify the amount of DR5 in the IP samples relative to the input lysate. [file elife-52291-fig1-data2.zip › Figure 1 - Source Data 2/Westerns for MPZ IP/Rep 2 and 3/2019-12-14 antiGAPDH-red antiGFP-green HCT116 MPZ vs GFP IP-2.tif]

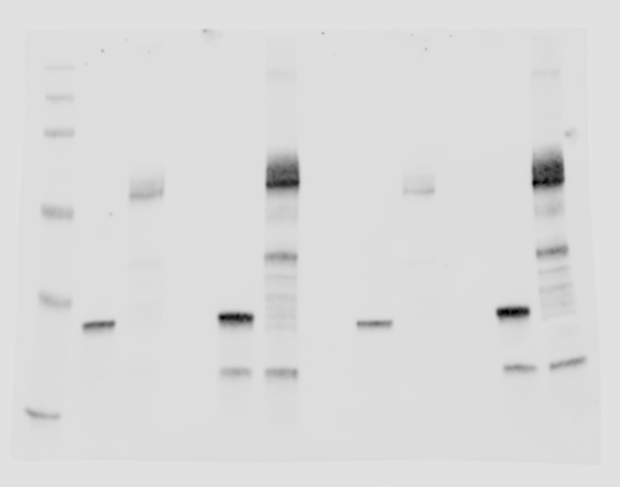

Supplement: Figure 1—source data 2. — This zip archive contains images of the Western blots and measurements used to quantify the amount of DR5 in the IP samples relative to the input lysate. [file elife-52291-fig1-data2.zip › Figure 1 - Source Data 2/Westerns for MPZ IP/Rep 2 and 3/2019-12-12 antiGFP HCT116 MPZ vs GFP IP.tif]

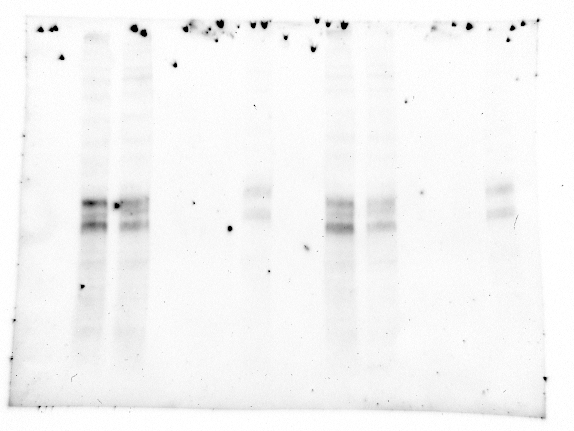

Supplement: Figure 1—source data 2. — This zip archive contains images of the Western blots and measurements used to quantify the amount of DR5 in the IP samples relative to the input lysate. [file elife-52291-fig1-data2.zip › Figure 1 - Source Data 2/Westerns for MPZ IP/Rep 2 and 3/2019-12-12 HCT116 MPZ-GFP IP antiDR5 Exposure_3000.0sec.tif]

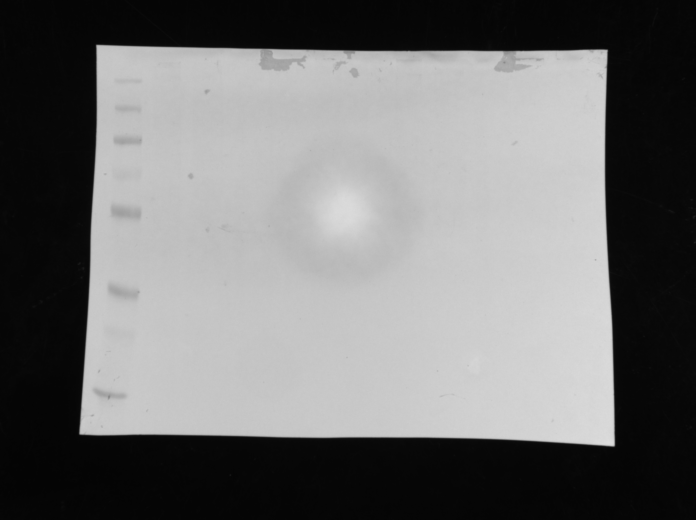

Supplement: Figure 1—source data 2. — This zip archive contains images of the Western blots and measurements used to quantify the amount of DR5 in the IP samples relative to the input lysate. [file elife-52291-fig1-data2.zip › Figure 1 - Source Data 2/Westerns for MPZ IP/Rep 2 and 3/2019-12-12 ladder HCT116 MPZ-GFP IP antiDR5 Exposure_3000.0sec.tif]

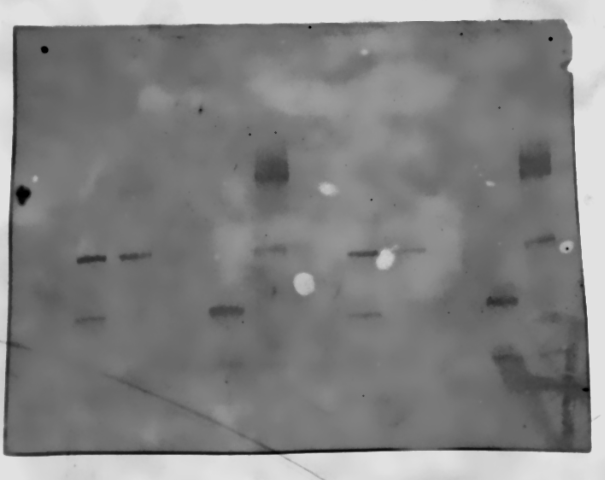

Supplement: Figure 1—source data 2. — This zip archive contains images of the Western blots and measurements used to quantify the amount of DR5 in the IP samples relative to the input lysate. [file elife-52291-fig1-data2.zip › Figure 1 - Source Data 2/Westerns for MPZ IP/Rep 2 and 3/2019-12-14 antiGAPDH HCT116 MPZ vs GFP IP.tif]

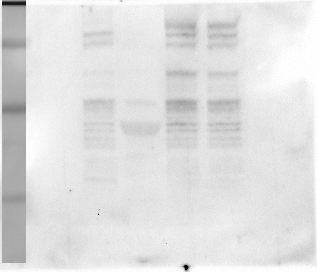

Supplement: Figure 1—source data 2. — This zip archive contains images of the Western blots and measurements used to quantify the amount of DR5 in the IP samples relative to the input lysate. [file elife-52291-fig1-data2.zip › Figure 1 - Source Data 2/Westerns for MPZ IP/Rep 1 - Figure 1G/2018-02-28 antiC8-MBL MPZ GFP IP HCT116_Exposure_180.0sec w ladder-1.tif]

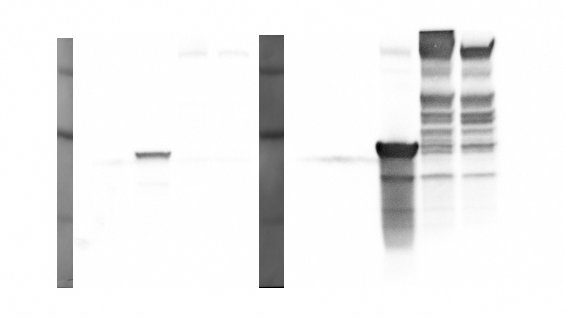

Supplement: Figure 1—source data 2. — This zip archive contains images of the Western blots and measurements used to quantify the amount of DR5 in the IP samples relative to the input lysate. [file elife-52291-fig1-data2.zip › Figure 1 - Source Data 2/Westerns for MPZ IP/Rep 1 - Figure 1G/2018-03-04 antiGFP MPZ GFP IP HCT116_Exposure_10.0sec.jpg]

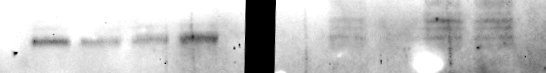

Supplement: Figure 1—source data 2. — This zip archive contains images of the Western blots and measurements used to quantify the amount of DR5 in the IP samples relative to the input lysate. [file elife-52291-fig1-data2.zip › Figure 1 - Source Data 2/Westerns for MPZ IP/Rep 1 - Figure 1G/2018-03-02 antiFADD MPZ GFP IP HCT116_Exposure_600.0sec-1.jpg]

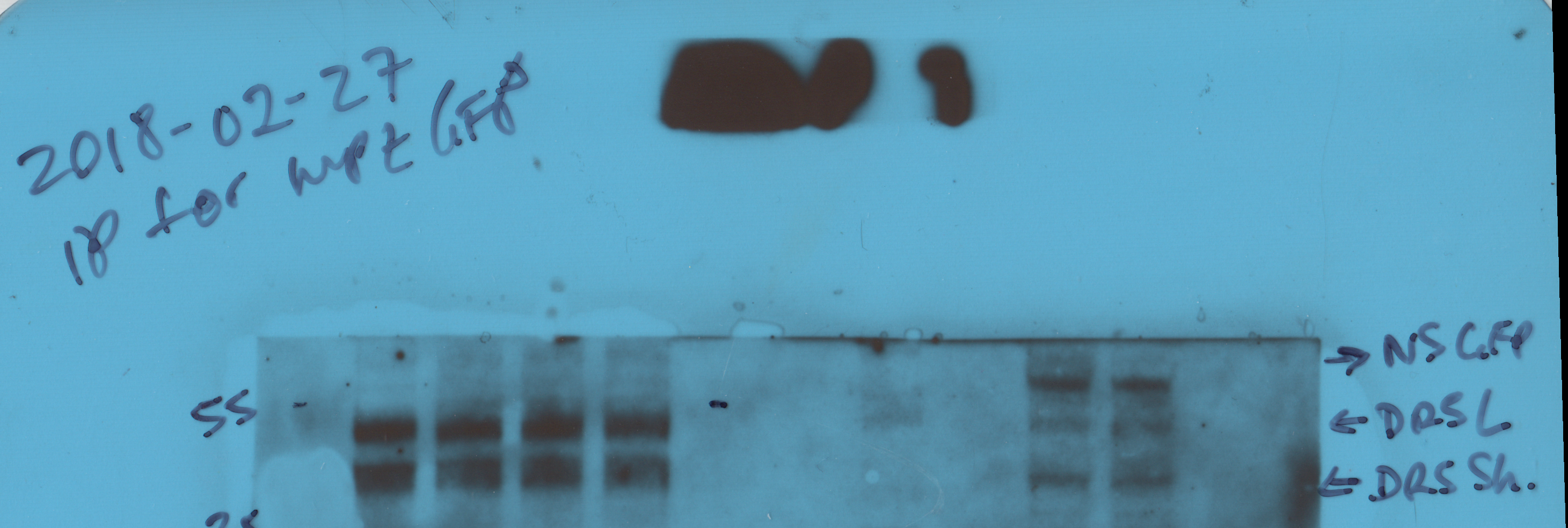

Supplement: Figure 1—source data 2. — This zip archive contains images of the Western blots and measurements used to quantify the amount of DR5 in the IP samples relative to the input lysate. [file elife-52291-fig1-data2.zip › Figure 1 - Source Data 2/Westerns for MPZ IP/Rep 1 - Figure 1G/2018-02-27 anti-DR5 on GFP IP MPZ traffo.tiff]

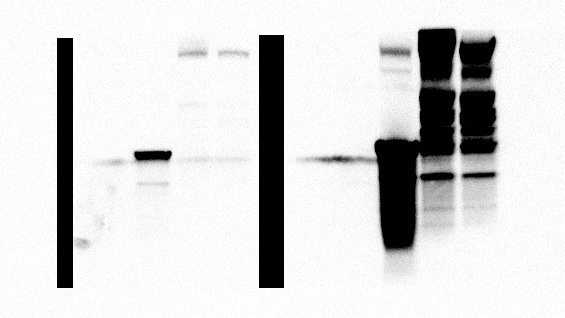

Supplement: Figure 1—source data 2. — This zip archive contains images of the Western blots and measurements used to quantify the amount of DR5 in the IP samples relative to the input lysate. [file elife-52291-fig1-data2.zip › Figure 1 - Source Data 2/Westerns for MPZ IP/Rep 1 - Figure 1G/2018-03-04 antiGFP MPZ GFP IP HCT116_for input.jpg]

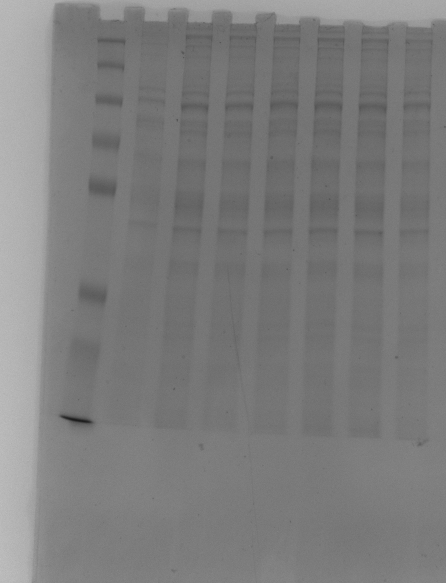

Supplement: Figure 1—source data 5. — This zip archive contains the measured luminescent units for caspase glo 8 activity shown in Figure 1—figure supplement 1E and the tif file of the Coomassie blue-stained gel used to normalize lysate concentrations. [file elife-52291-fig1-data5.zip › Figure 1 - Source Data 5/Source Data Fig 1S1E - Coomassie MPZ time course.tif]

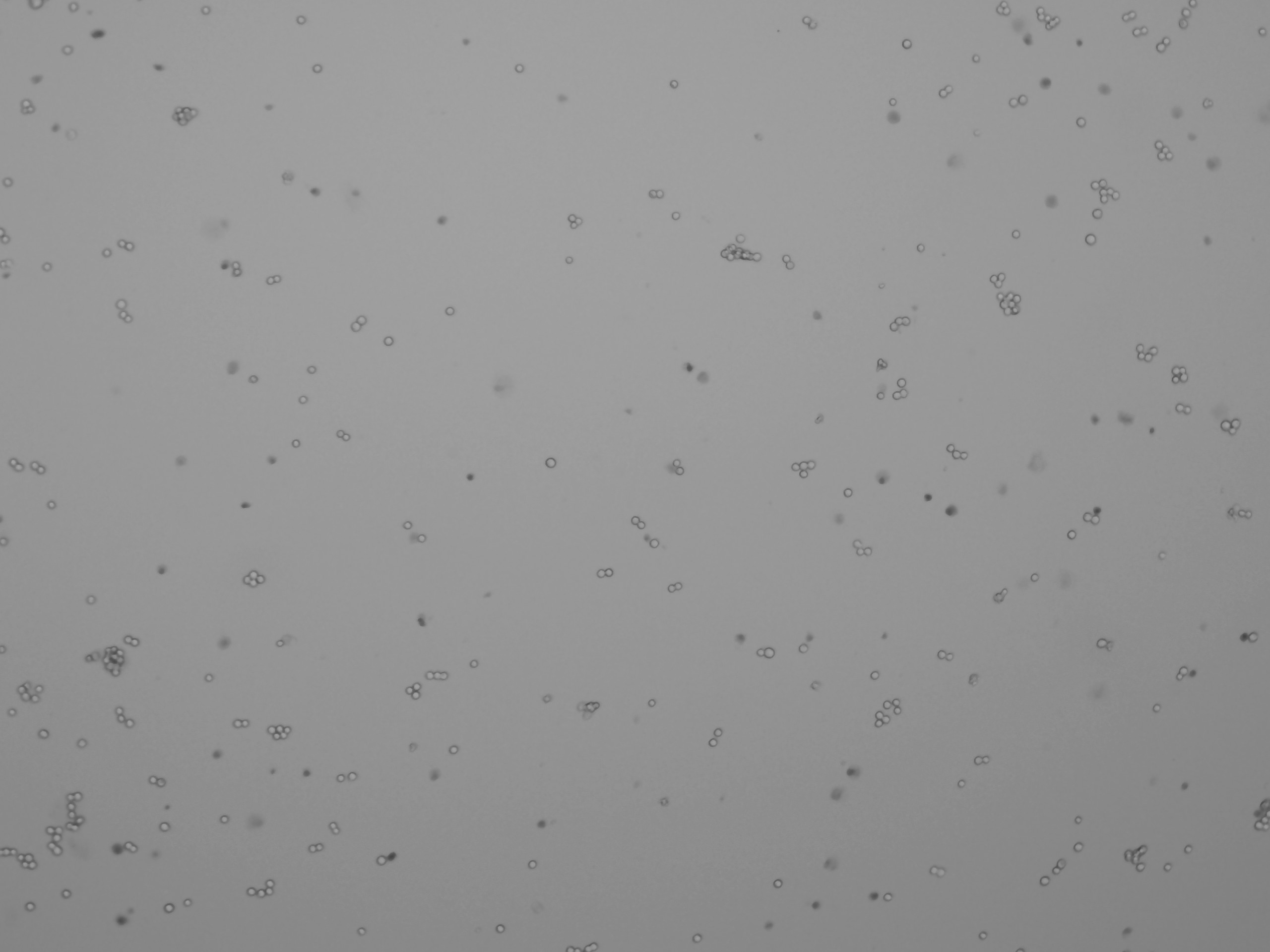

Supplement: Figure 1—source data 6. — This zip archive contains the qPCR analysis from CHOP expression in Figure 1—figure supplement 2B, and brightfield images of Trypan Blue staining measured on the Countess II for n = 3 biological replicates, summarized in Figure 1—figure supplement 2D. [file elife-52291-fig1-data6.zip › Figure 1 - Source Data 6/Source Data Fig 1S2D - Trypan Blue for CHOP expression/hct chop 500_BF.jpg]

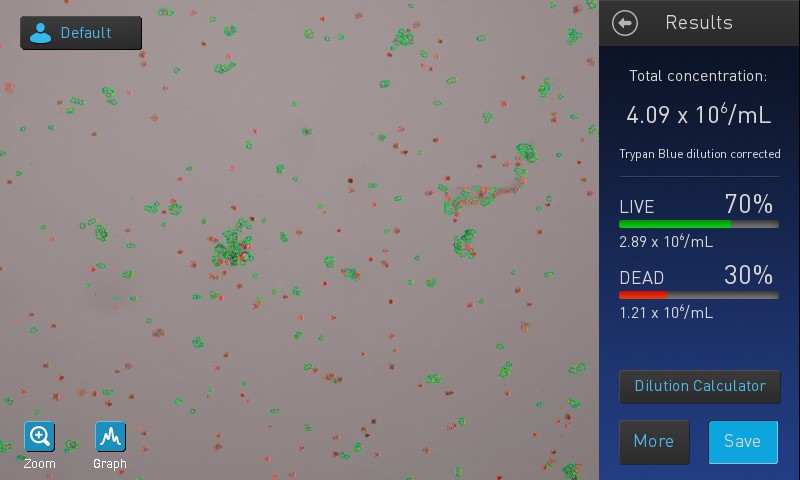

Supplement: Figure 1—source data 6. — This zip archive contains the qPCR analysis from CHOP expression in Figure 1—figure supplement 2B, and brightfield images of Trypan Blue staining measured on the Countess II for n = 3 biological replicates, summarized in Figure 1—figure supplement 2D. [file elife-52291-fig1-data6.zip › Figure 1 - Source Data 6/Source Data Fig 1S2D - Trypan Blue for CHOP expression/20190523 hct mpz rep 3.jpg]

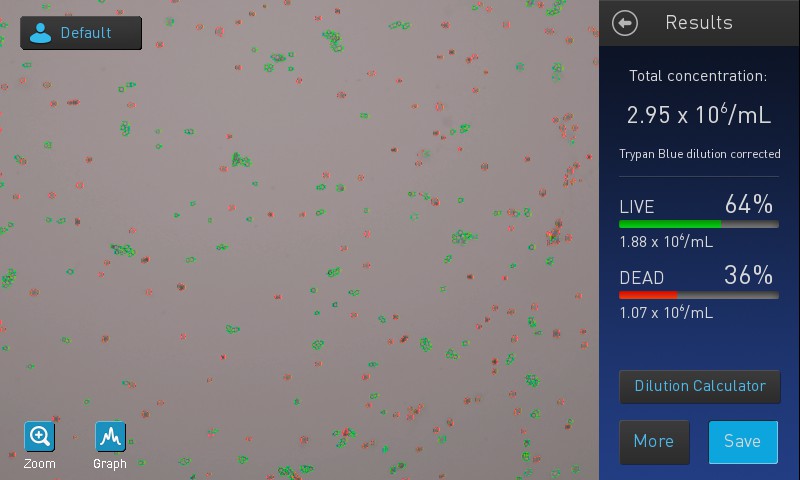

Supplement: Figure 1—source data 6. — This zip archive contains the qPCR analysis from CHOP expression in Figure 1—figure supplement 2B, and brightfield images of Trypan Blue staining measured on the Countess II for n = 3 biological replicates, summarized in Figure 1—figure supplement 2D. [file elife-52291-fig1-data6.zip › Figure 1 - Source Data 6/Source Data Fig 1S2D - Trypan Blue for CHOP expression/20190523 hct mpz rep 2.jpg]

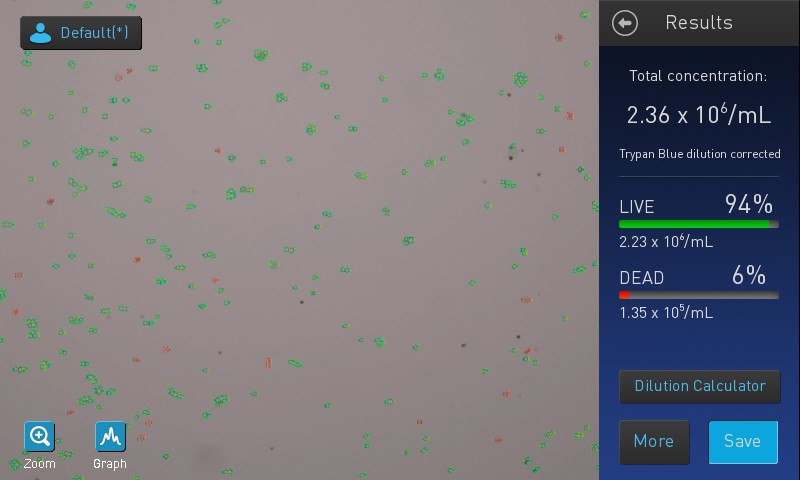

Supplement: Figure 1—source data 6. — This zip archive contains the qPCR analysis from CHOP expression in Figure 1—figure supplement 2B, and brightfield images of Trypan Blue staining measured on the Countess II for n = 3 biological replicates, summarized in Figure 1—figure supplement 2D. [file elife-52291-fig1-data6.zip › Figure 1 - Source Data 6/Source Data Fig 1S2D - Trypan Blue for CHOP expression/hct chop 62.jpg]

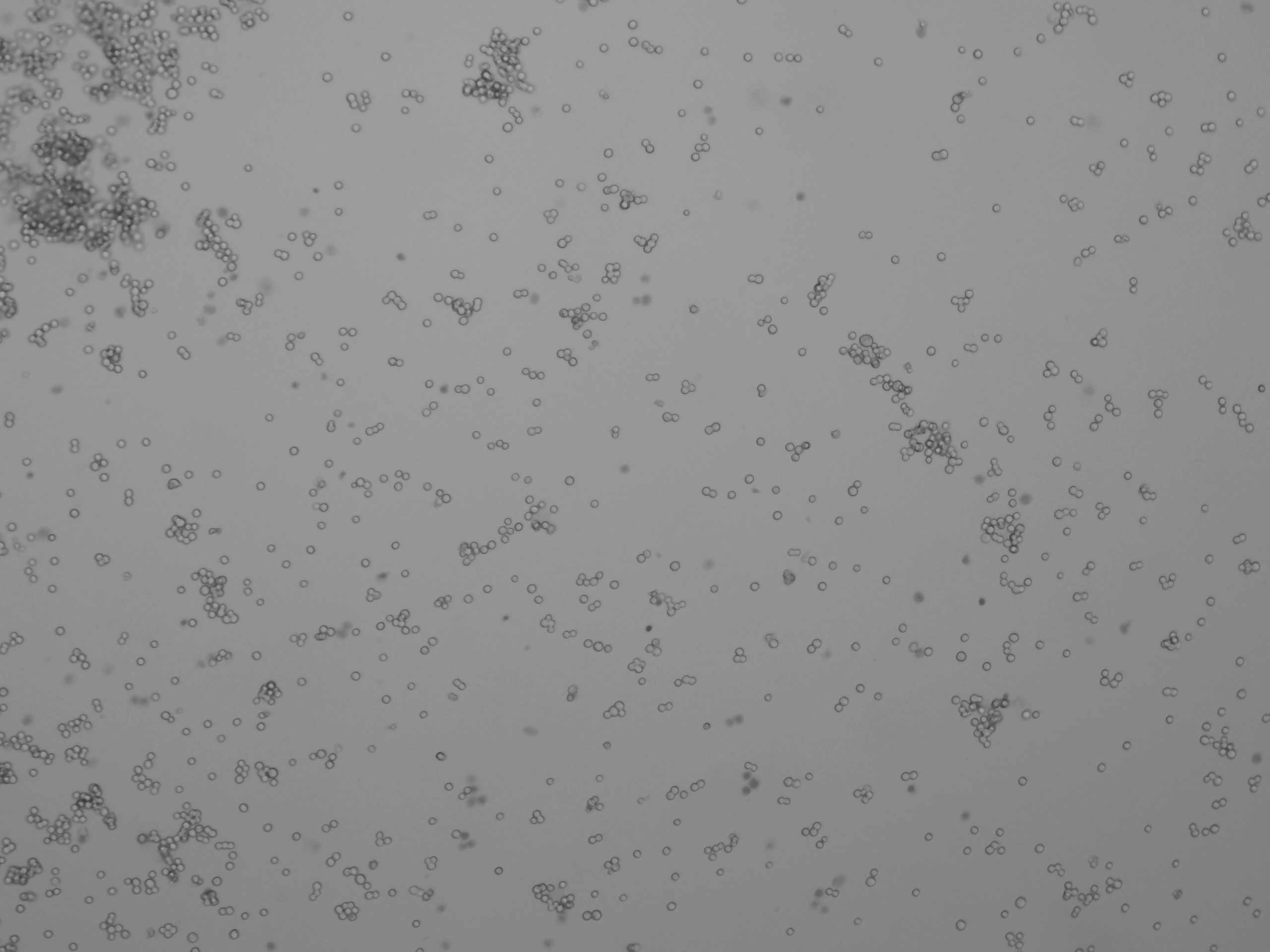

Supplement: Figure 1—source data 6. — This zip archive contains the qPCR analysis from CHOP expression in Figure 1—figure supplement 2B, and brightfield images of Trypan Blue staining measured on the Countess II for n = 3 biological replicates, summarized in Figure 1—figure supplement 2D. [file elife-52291-fig1-data6.zip › Figure 1 - Source Data 6/Source Data Fig 1S2D - Trypan Blue for CHOP expression/20190523 hct chop 250 rep 3_BF.jpg]

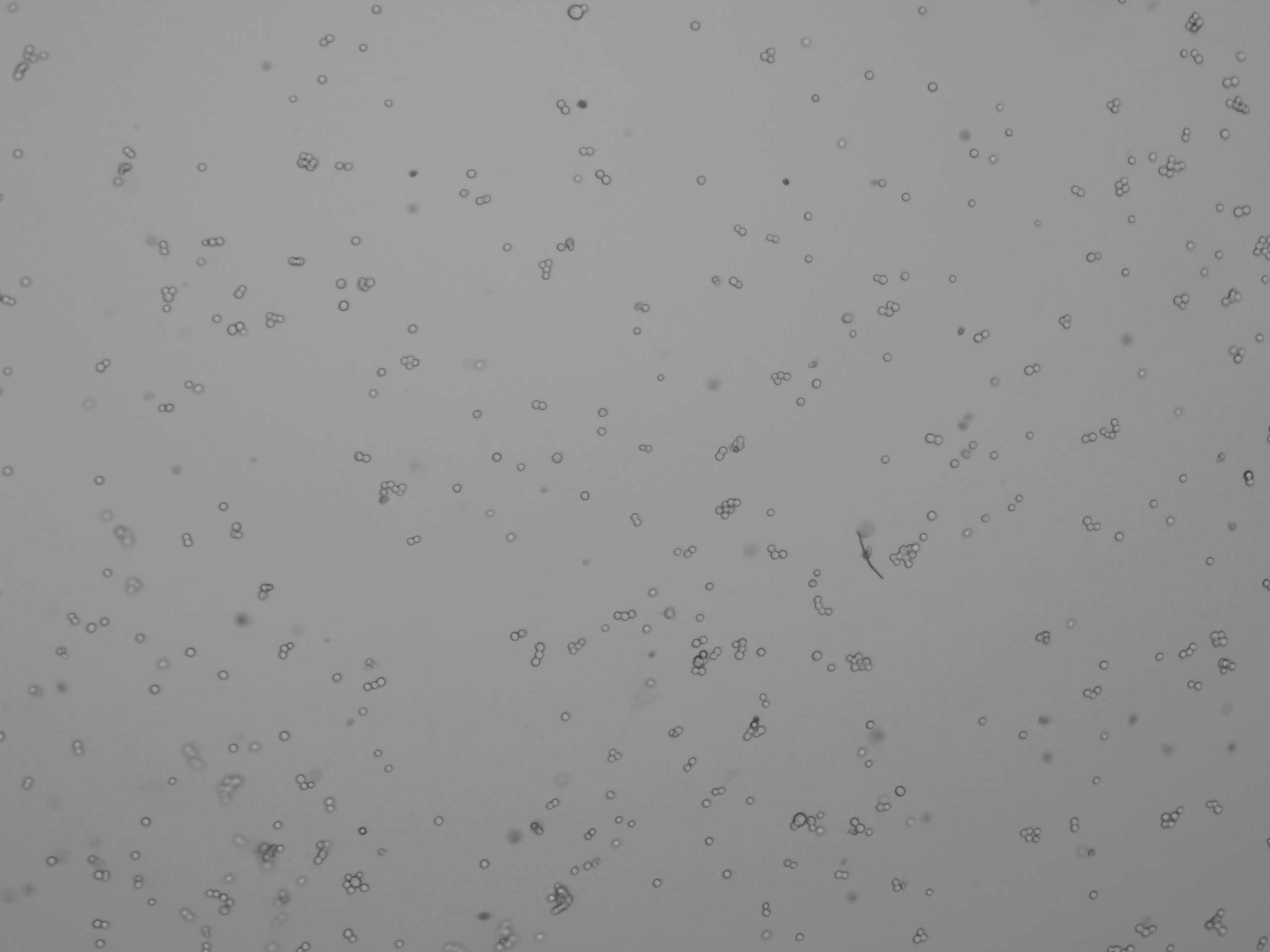

Supplement: Figure 1—source data 6. — This zip archive contains the qPCR analysis from CHOP expression in Figure 1—figure supplement 2B, and brightfield images of Trypan Blue staining measured on the Countess II for n = 3 biological replicates, summarized in Figure 1—figure supplement 2D. [file elife-52291-fig1-data6.zip › Figure 1 - Source Data 6/Source Data Fig 1S2D - Trypan Blue for CHOP expression/20190523 hct chop 1000 rep 2_BF.jpg]

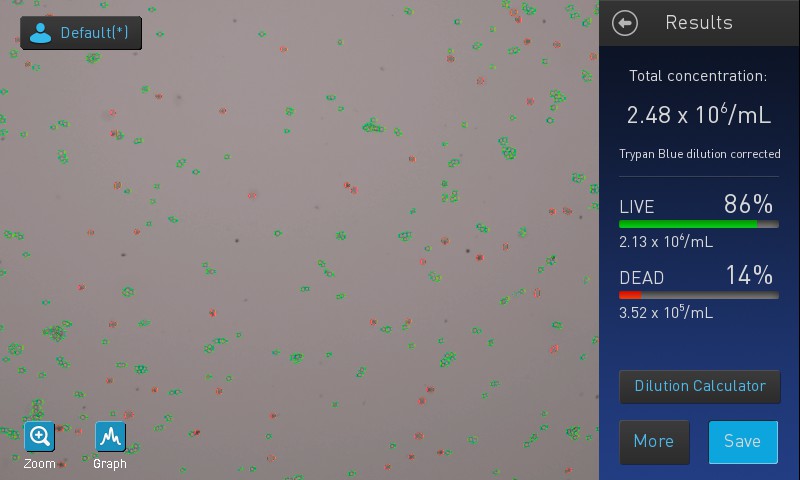

Supplement: Figure 1—source data 6. — This zip archive contains the qPCR analysis from CHOP expression in Figure 1—figure supplement 2B, and brightfield images of Trypan Blue staining measured on the Countess II for n = 3 biological replicates, summarized in Figure 1—figure supplement 2D. [file elife-52291-fig1-data6.zip › Figure 1 - Source Data 6/Source Data Fig 1S2D - Trypan Blue for CHOP expression/hct chop 500.jpg]

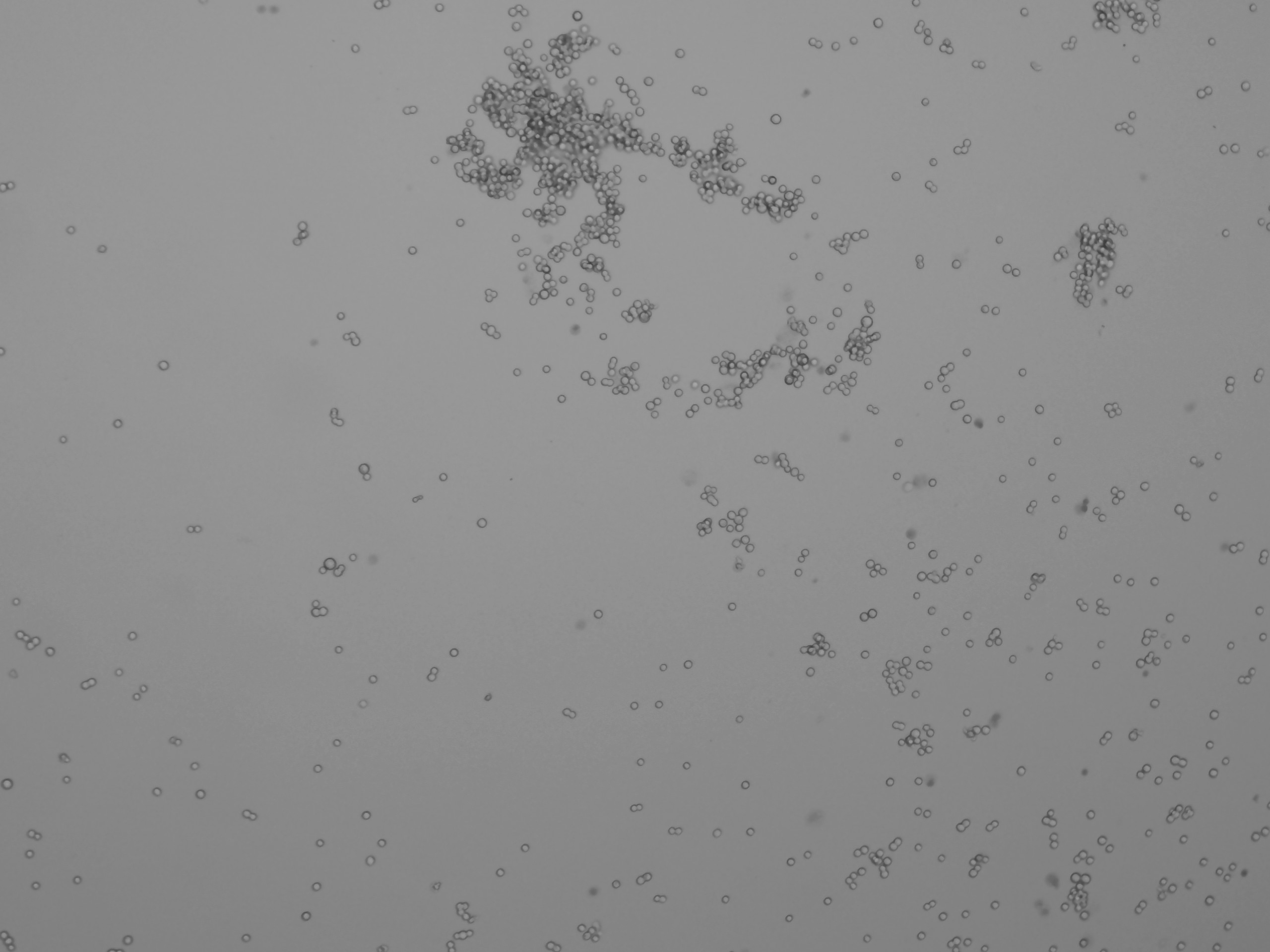

Supplement: Figure 1—source data 6. — This zip archive contains the qPCR analysis from CHOP expression in Figure 1—figure supplement 2B, and brightfield images of Trypan Blue staining measured on the Countess II for n = 3 biological replicates, summarized in Figure 1—figure supplement 2D. [file elife-52291-fig1-data6.zip › Figure 1 - Source Data 6/Source Data Fig 1S2D - Trypan Blue for CHOP expression/20190523 hct chop 125 rep 2_BF.jpg]

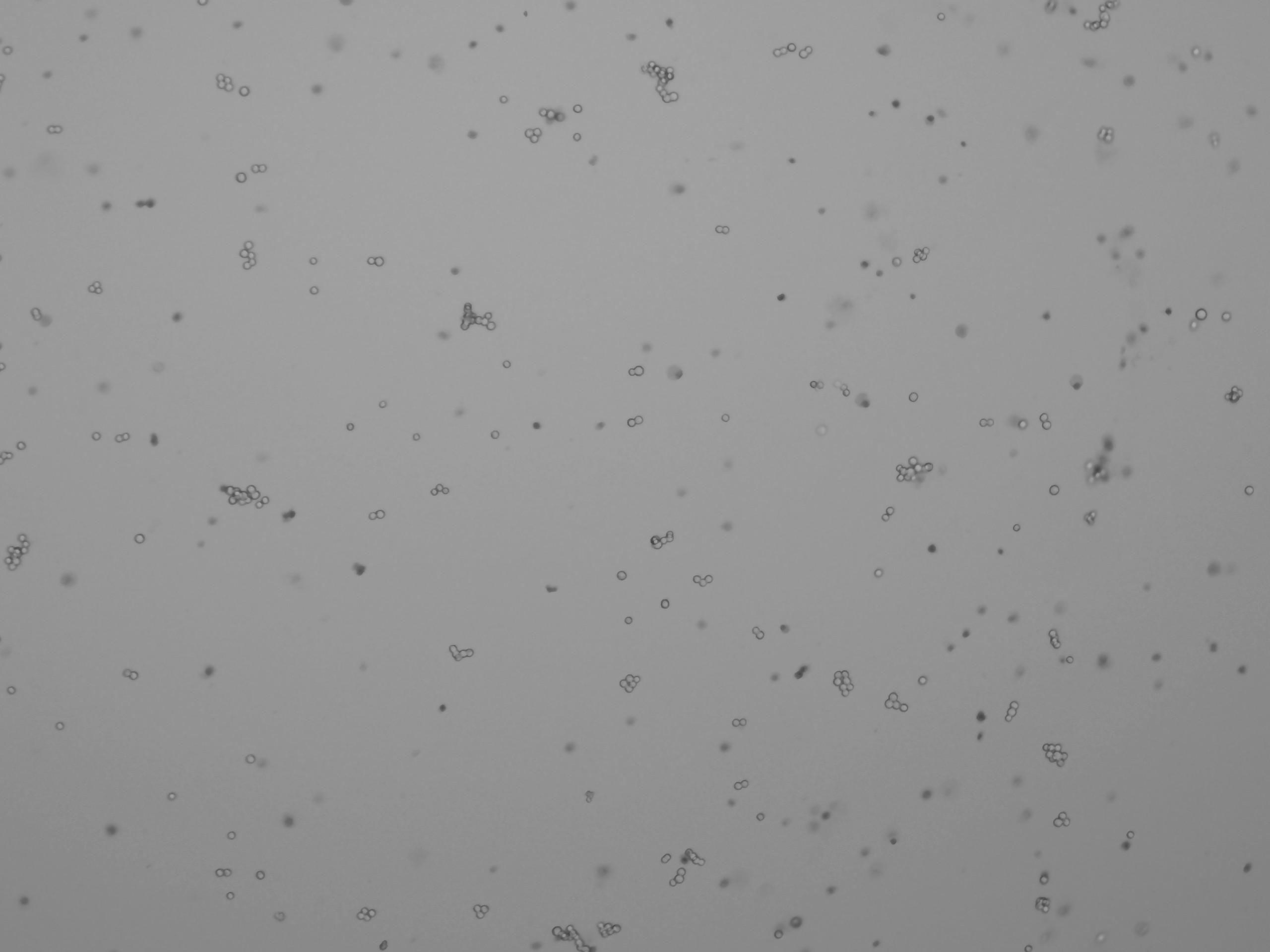

Supplement: Figure 1—source data 6. — This zip archive contains the qPCR analysis from CHOP expression in Figure 1—figure supplement 2B, and brightfield images of Trypan Blue staining measured on the Countess II for n = 3 biological replicates, summarized in Figure 1—figure supplement 2D. [file elife-52291-fig1-data6.zip › Figure 1 - Source Data 6/Source Data Fig 1S2D - Trypan Blue for CHOP expression/20190523 hct mpz rep 2_BF.jpg]

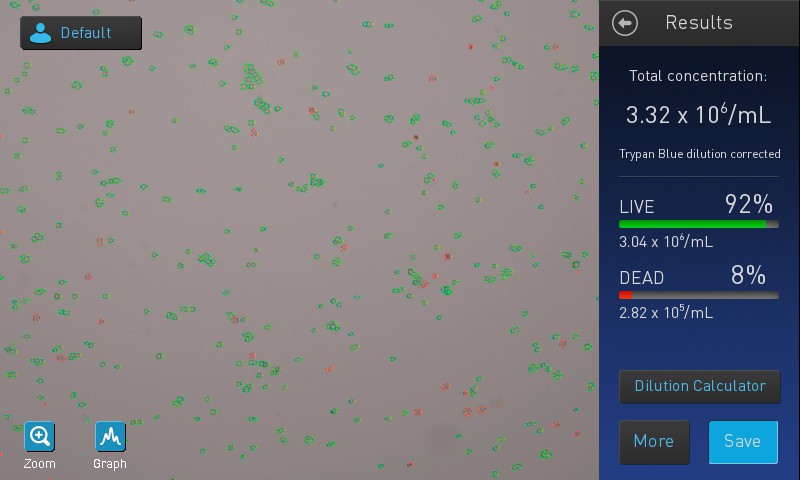

Supplement: Figure 1—source data 6. — This zip archive contains the qPCR analysis from CHOP expression in Figure 1—figure supplement 2B, and brightfield images of Trypan Blue staining measured on the Countess II for n = 3 biological replicates, summarized in Figure 1—figure supplement 2D. [file elife-52291-fig1-data6.zip › Figure 1 - Source Data 6/Source Data Fig 1S2D - Trypan Blue for CHOP expression/20190523 hct chop 1000 rep 1.jpg]

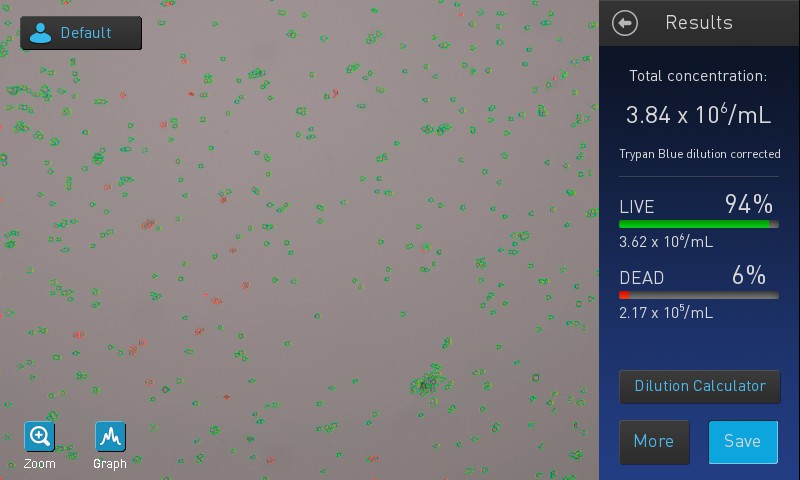

Supplement: Figure 1—source data 6. — This zip archive contains the qPCR analysis from CHOP expression in Figure 1—figure supplement 2B, and brightfield images of Trypan Blue staining measured on the Countess II for n = 3 biological replicates, summarized in Figure 1—figure supplement 2D. [file elife-52291-fig1-data6.zip › Figure 1 - Source Data 6/Source Data Fig 1S2D - Trypan Blue for CHOP expression/20190523 hct empty rep 2.jpg]

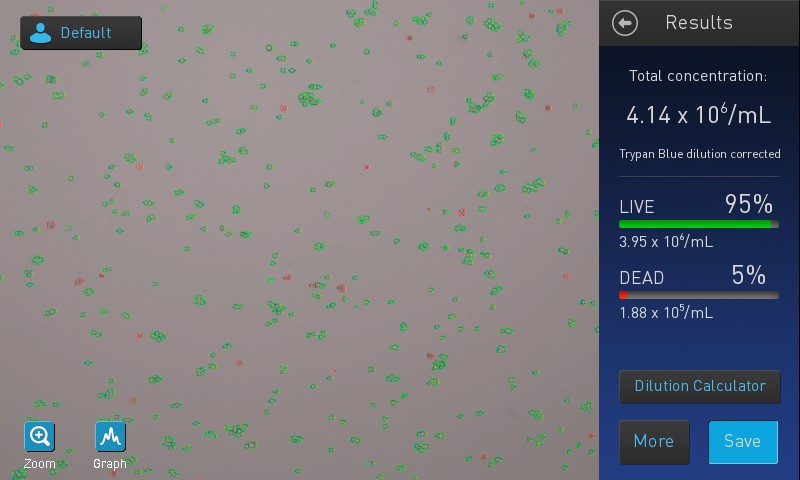

Supplement: Figure 1—source data 6. — This zip archive contains the qPCR analysis from CHOP expression in Figure 1—figure supplement 2B, and brightfield images of Trypan Blue staining measured on the Countess II for n = 3 biological replicates, summarized in Figure 1—figure supplement 2D. [file elife-52291-fig1-data6.zip › Figure 1 - Source Data 6/Source Data Fig 1S2D - Trypan Blue for CHOP expression/20190523 hct chop 1000 rep 3.jpg]

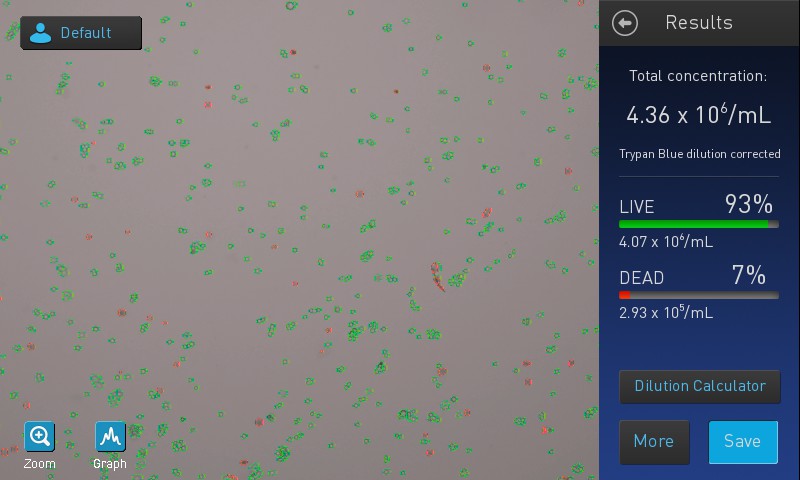

Supplement: Figure 1—source data 6. — This zip archive contains the qPCR analysis from CHOP expression in Figure 1—figure supplement 2B, and brightfield images of Trypan Blue staining measured on the Countess II for n = 3 biological replicates, summarized in Figure 1—figure supplement 2D. [file elife-52291-fig1-data6.zip › Figure 1 - Source Data 6/Source Data Fig 1S2D - Trypan Blue for CHOP expression/20190523 hct chop 1000 rep 2.jpg]

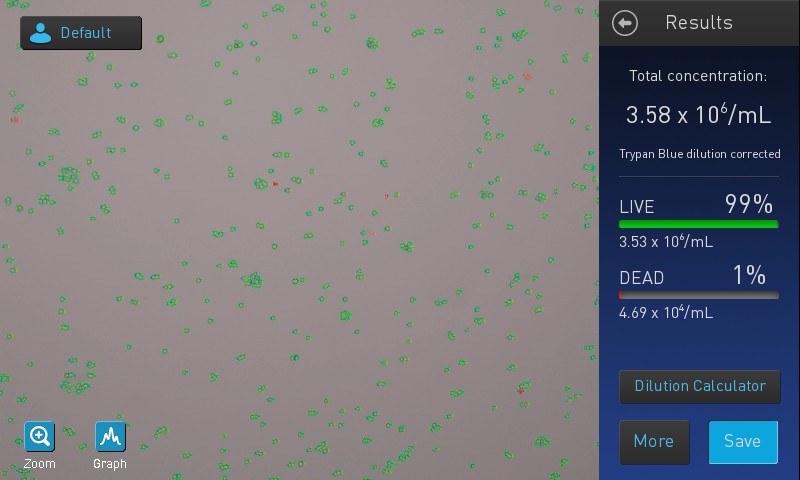

Supplement: Figure 1—source data 6. — This zip archive contains the qPCR analysis from CHOP expression in Figure 1—figure supplement 2B, and brightfield images of Trypan Blue staining measured on the Countess II for n = 3 biological replicates, summarized in Figure 1—figure supplement 2D. [file elife-52291-fig1-data6.zip › Figure 1 - Source Data 6/Source Data Fig 1S2D - Trypan Blue for CHOP expression/20190523 hct empty rep 3.jpg]

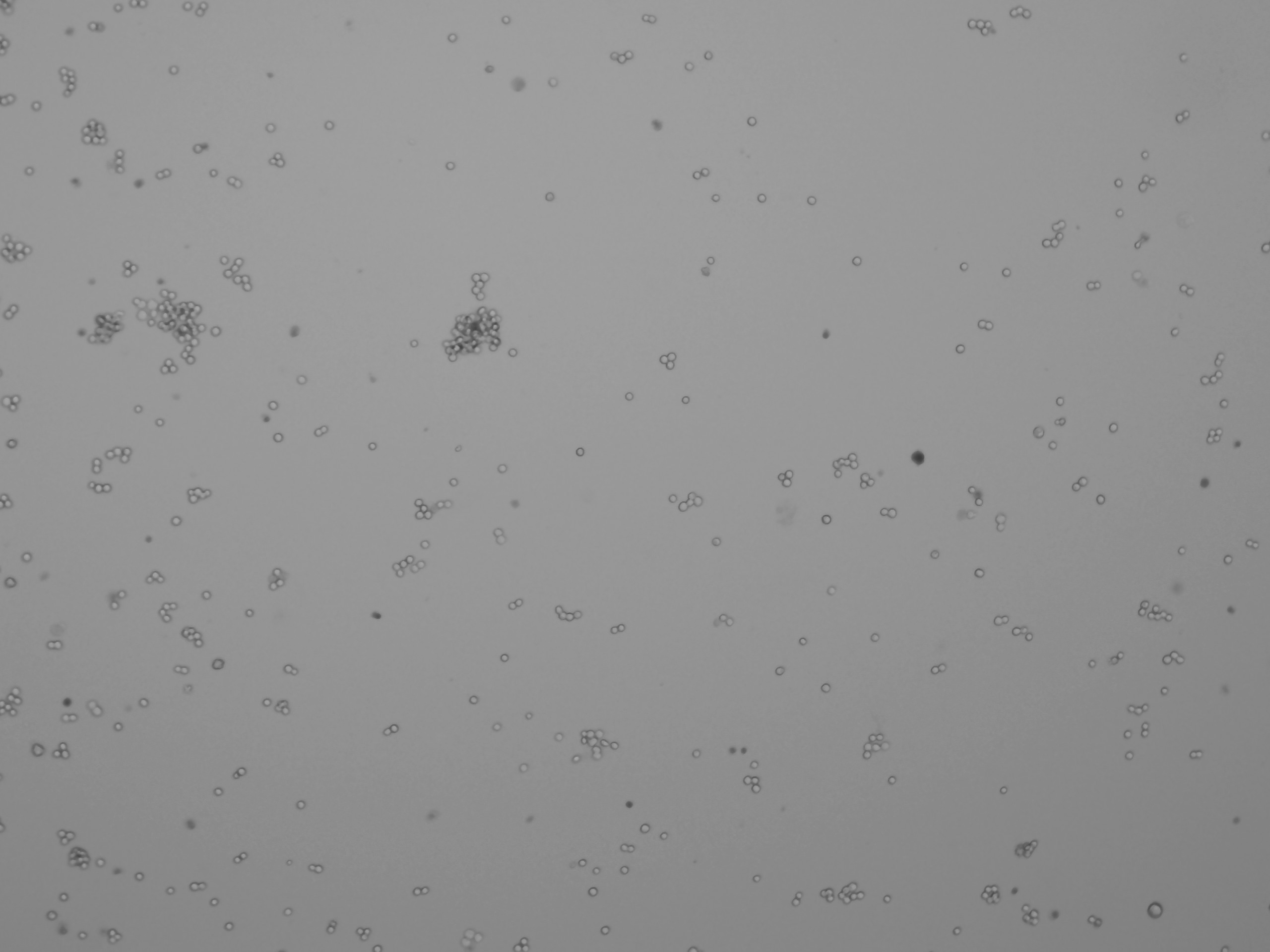

Supplement: Figure 1—source data 6. — This zip archive contains the qPCR analysis from CHOP expression in Figure 1—figure supplement 2B, and brightfield images of Trypan Blue staining measured on the Countess II for n = 3 biological replicates, summarized in Figure 1—figure supplement 2D. [file elife-52291-fig1-data6.zip › Figure 1 - Source Data 6/Source Data Fig 1S2D - Trypan Blue for CHOP expression/hct chop 125_BF.jpg]

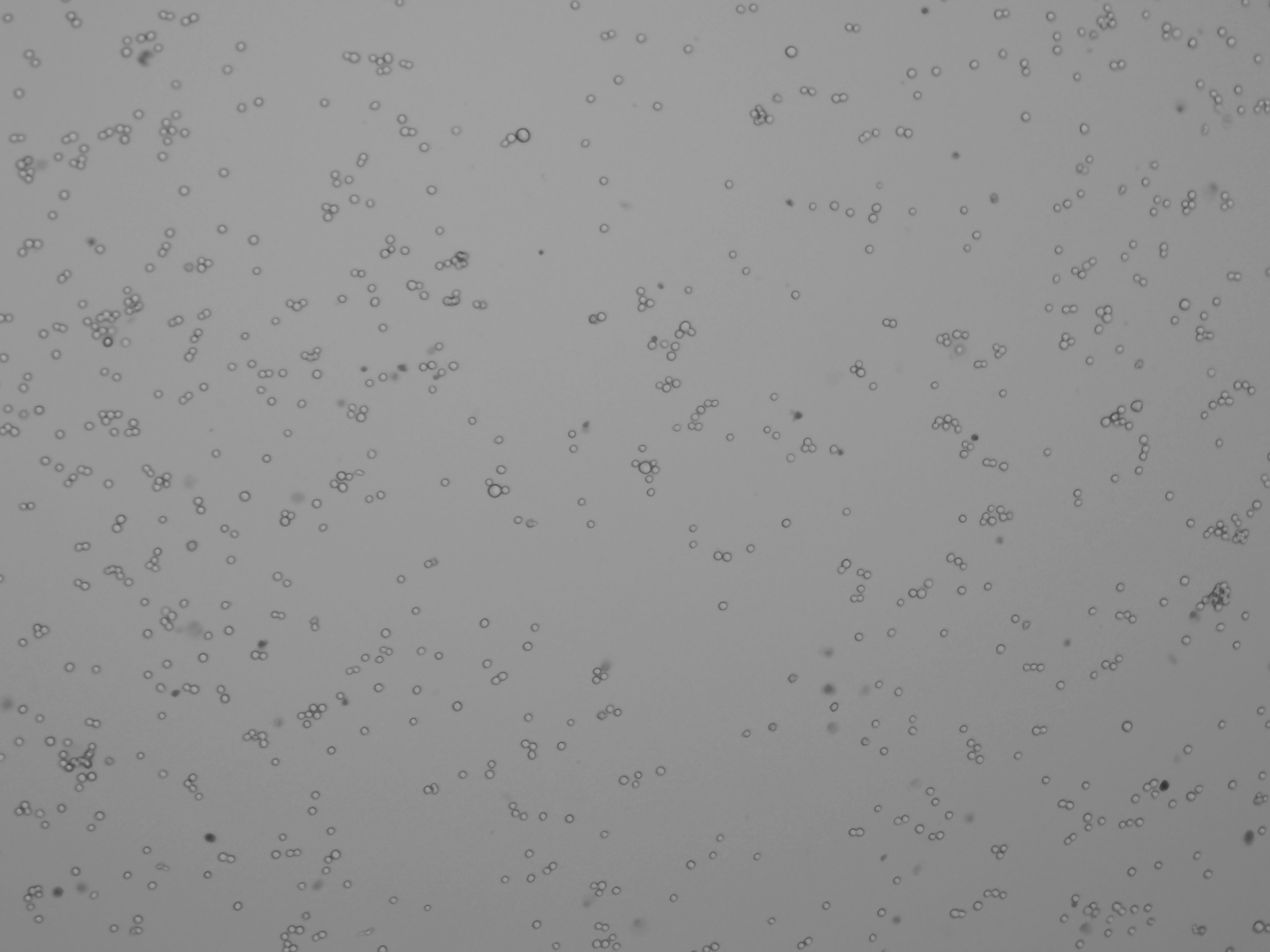

Supplement: Figure 1—source data 6. — This zip archive contains the qPCR analysis from CHOP expression in Figure 1—figure supplement 2B, and brightfield images of Trypan Blue staining measured on the Countess II for n = 3 biological replicates, summarized in Figure 1—figure supplement 2D. [file elife-52291-fig1-data6.zip › Figure 1 - Source Data 6/Source Data Fig 1S2D - Trypan Blue for CHOP expression/hct empty_BF.jpg]

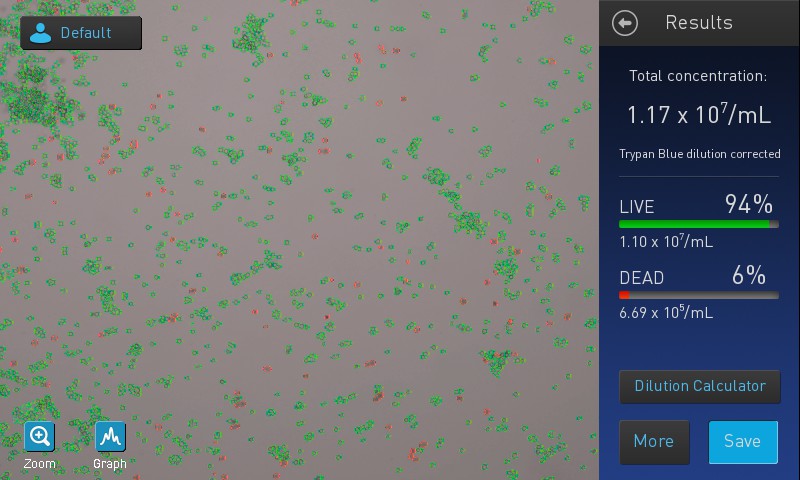

Supplement: Figure 1—source data 6. — This zip archive contains the qPCR analysis from CHOP expression in Figure 1—figure supplement 2B, and brightfield images of Trypan Blue staining measured on the Countess II for n = 3 biological replicates, summarized in Figure 1—figure supplement 2D. [file elife-52291-fig1-data6.zip › Figure 1 - Source Data 6/Source Data Fig 1S2D - Trypan Blue for CHOP expression/20190523 hct chop 250 rep 3.jpg]

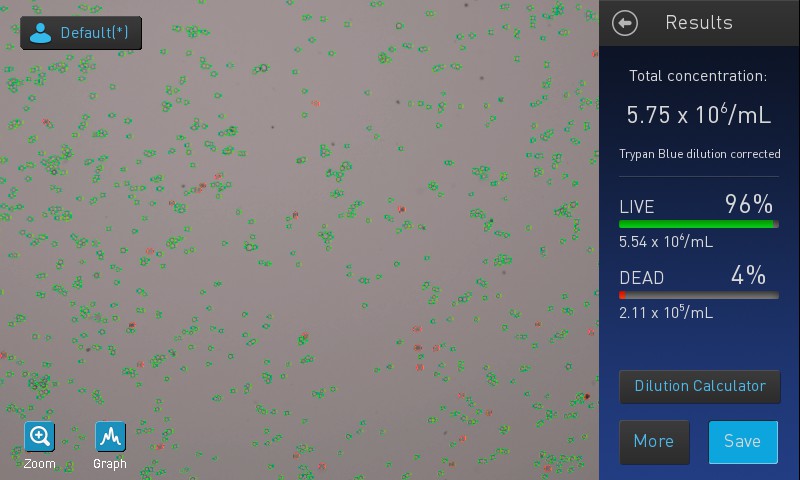

Supplement: Figure 1—source data 6. — This zip archive contains the qPCR analysis from CHOP expression in Figure 1—figure supplement 2B, and brightfield images of Trypan Blue staining measured on the Countess II for n = 3 biological replicates, summarized in Figure 1—figure supplement 2D. [file elife-52291-fig1-data6.zip › Figure 1 - Source Data 6/Source Data Fig 1S2D - Trypan Blue for CHOP expression/hct empty.jpg]

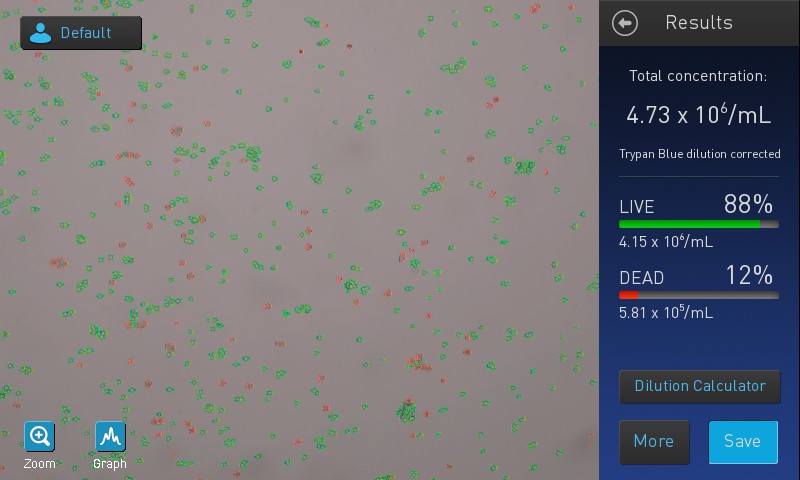

Supplement: Figure 1—source data 6. — This zip archive contains the qPCR analysis from CHOP expression in Figure 1—figure supplement 2B, and brightfield images of Trypan Blue staining measured on the Countess II for n = 3 biological replicates, summarized in Figure 1—figure supplement 2D. [file elife-52291-fig1-data6.zip › Figure 1 - Source Data 6/Source Data Fig 1S2D - Trypan Blue for CHOP expression/20190523 hct chop 250 rep 2.jpg]

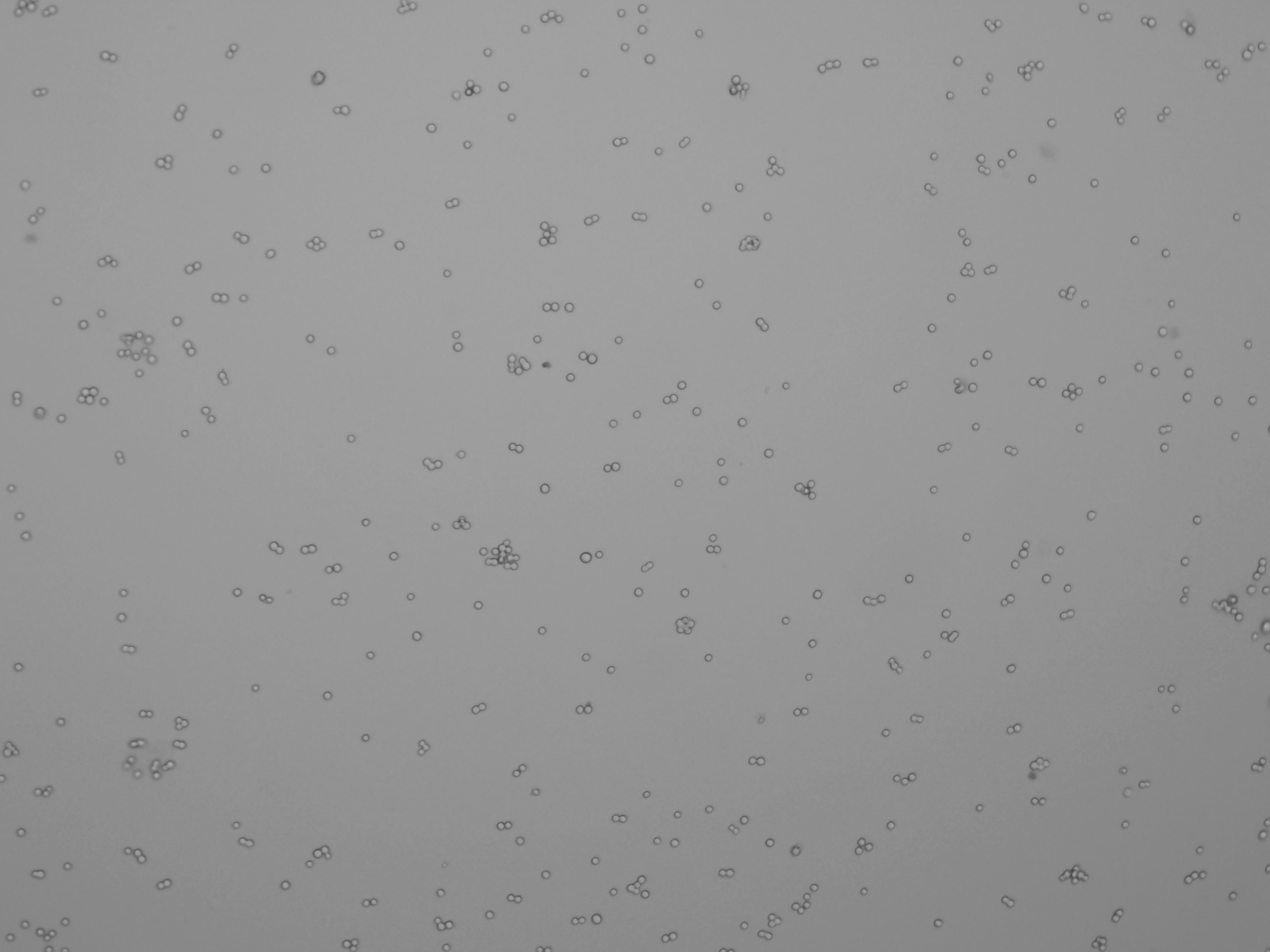

Supplement: Figure 1—source data 6. — This zip archive contains the qPCR analysis from CHOP expression in Figure 1—figure supplement 2B, and brightfield images of Trypan Blue staining measured on the Countess II for n = 3 biological replicates, summarized in Figure 1—figure supplement 2D. [file elife-52291-fig1-data6.zip › Figure 1 - Source Data 6/Source Data Fig 1S2D - Trypan Blue for CHOP expression/20190523 hct empty rep 3_BF.jpg]

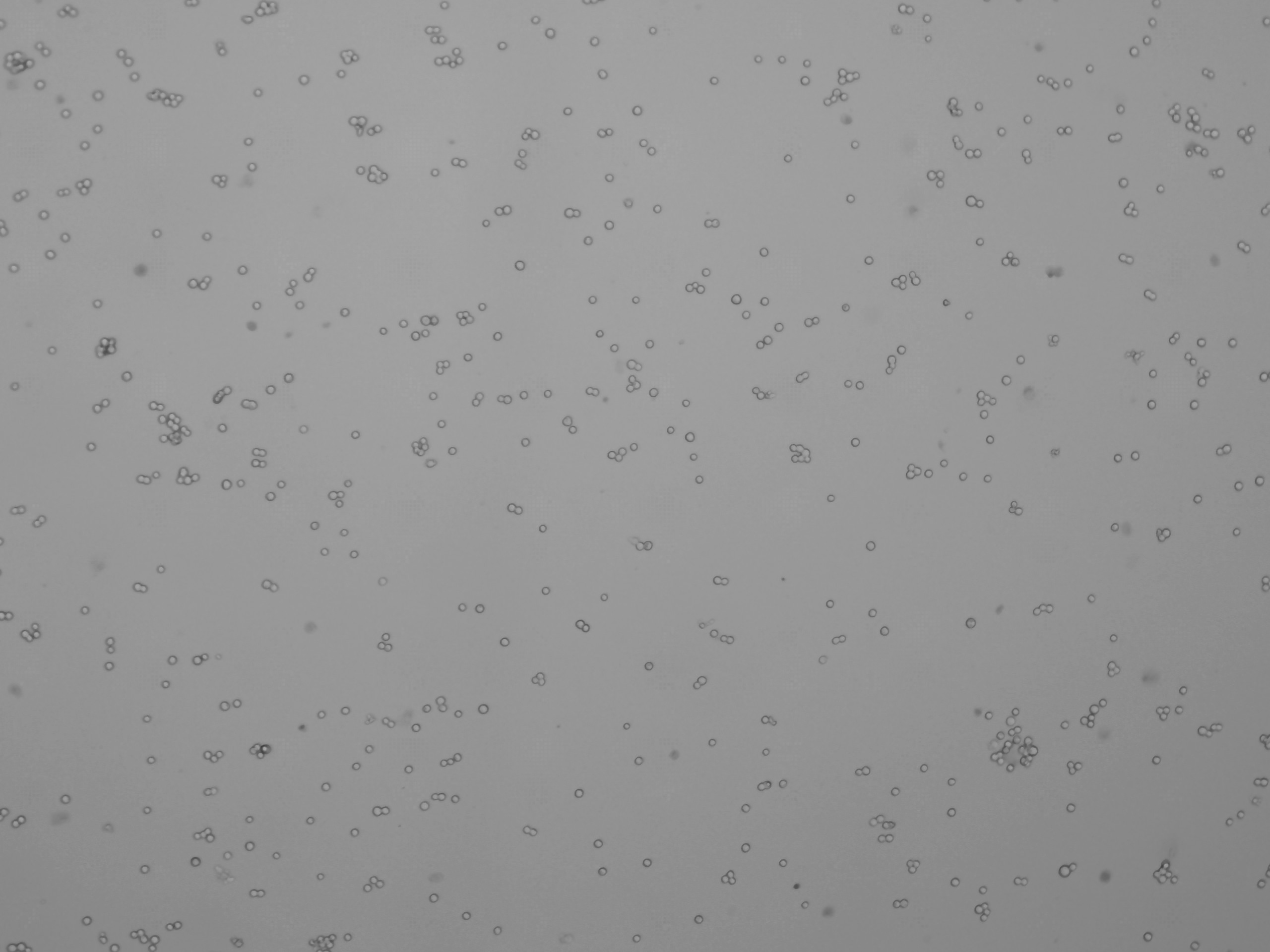

Supplement: Figure 1—source data 6. — This zip archive contains the qPCR analysis from CHOP expression in Figure 1—figure supplement 2B, and brightfield images of Trypan Blue staining measured on the Countess II for n = 3 biological replicates, summarized in Figure 1—figure supplement 2D. [file elife-52291-fig1-data6.zip › Figure 1 - Source Data 6/Source Data Fig 1S2D - Trypan Blue for CHOP expression/20190523 hct chop 500 rep 2_BF.jpg]

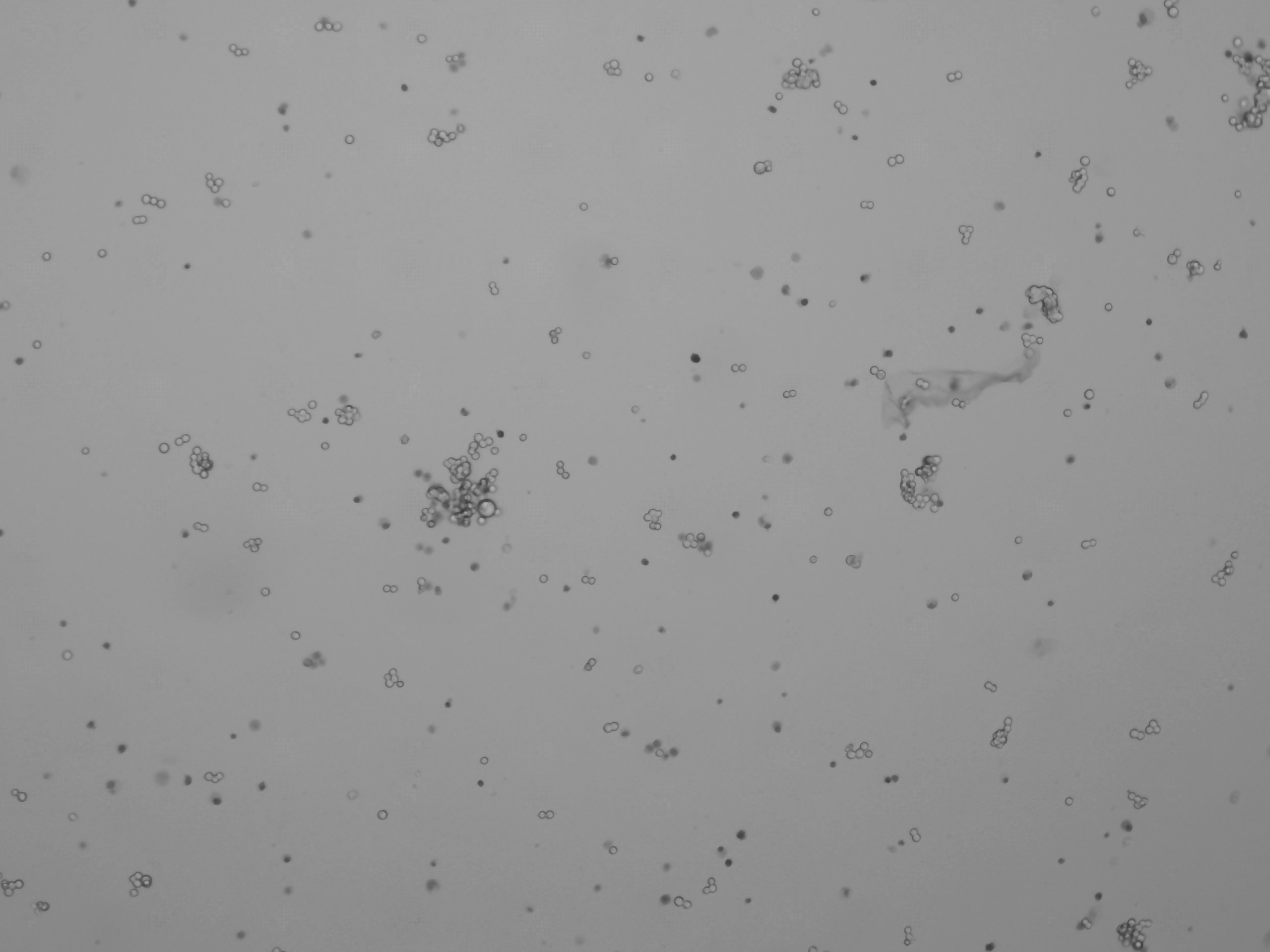

Supplement: Figure 1—source data 6. — This zip archive contains the qPCR analysis from CHOP expression in Figure 1—figure supplement 2B, and brightfield images of Trypan Blue staining measured on the Countess II for n = 3 biological replicates, summarized in Figure 1—figure supplement 2D. [file elife-52291-fig1-data6.zip › Figure 1 - Source Data 6/Source Data Fig 1S2D - Trypan Blue for CHOP expression/20190523 hct mpz rep 3_BF.jpg]

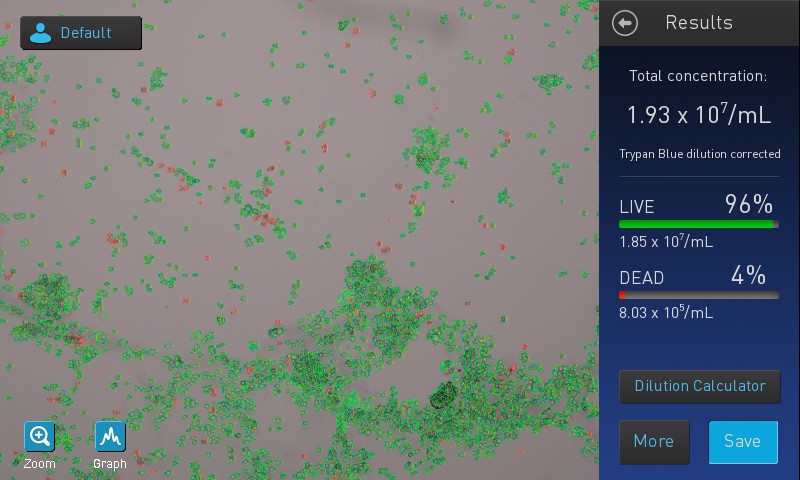

Supplement: Figure 1—source data 6. — This zip archive contains the qPCR analysis from CHOP expression in Figure 1—figure supplement 2B, and brightfield images of Trypan Blue staining measured on the Countess II for n = 3 biological replicates, summarized in Figure 1—figure supplement 2D. [file elife-52291-fig1-data6.zip › Figure 1 - Source Data 6/Source Data Fig 1S2D - Trypan Blue for CHOP expression/20190523 hct chop 125 rep 3.jpg]

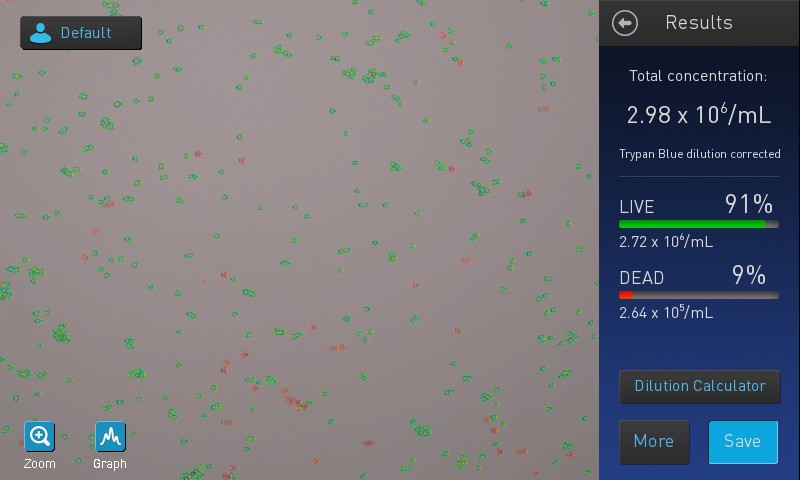

Supplement: Figure 1—source data 6. — This zip archive contains the qPCR analysis from CHOP expression in Figure 1—figure supplement 2B, and brightfield images of Trypan Blue staining measured on the Countess II for n = 3 biological replicates, summarized in Figure 1—figure supplement 2D. [file elife-52291-fig1-data6.zip › Figure 1 - Source Data 6/Source Data Fig 1S2D - Trypan Blue for CHOP expression/20190523 hct chop empty rep 3.jpg]

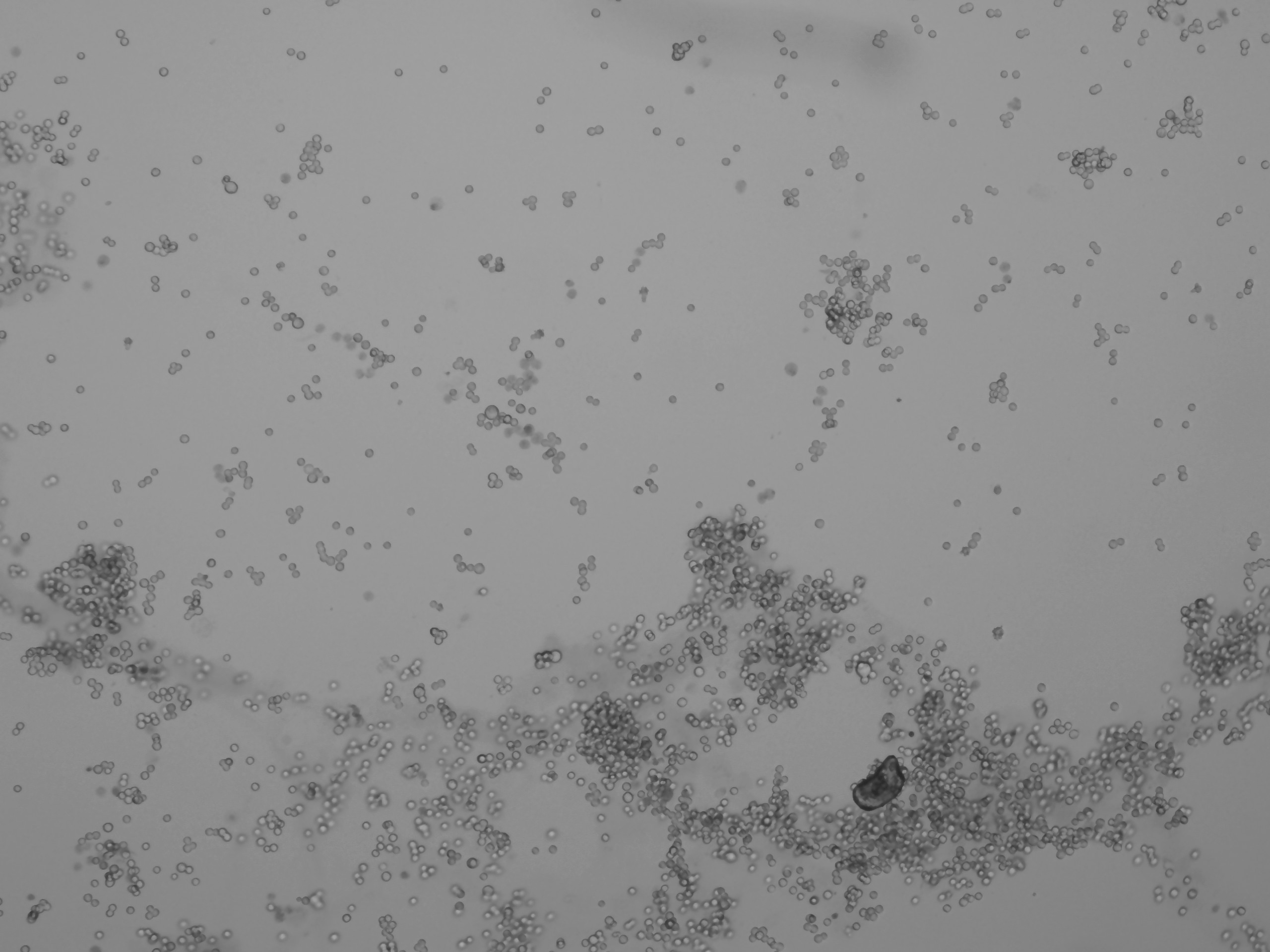

Supplement: Figure 1—source data 6. — This zip archive contains the qPCR analysis from CHOP expression in Figure 1—figure supplement 2B, and brightfield images of Trypan Blue staining measured on the Countess II for n = 3 biological replicates, summarized in Figure 1—figure supplement 2D. [file elife-52291-fig1-data6.zip › Figure 1 - Source Data 6/Source Data Fig 1S2D - Trypan Blue for CHOP expression/20190523 hct chop 125 rep 3_BF.jpg]

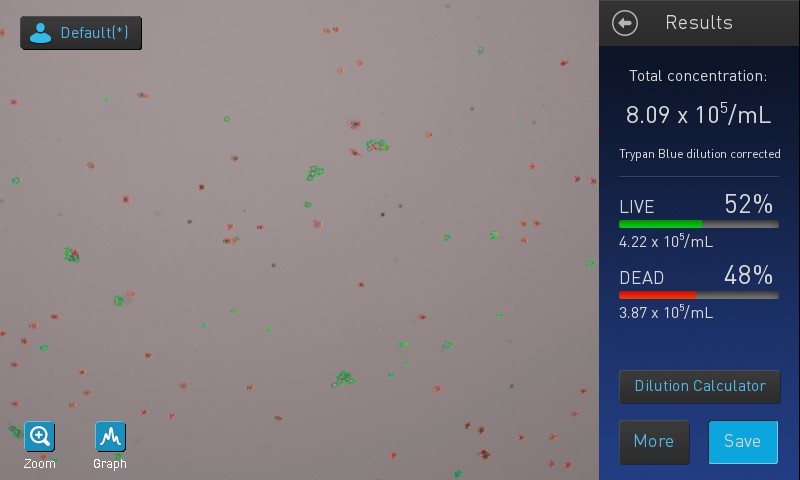

Supplement: Figure 1—source data 6. — This zip archive contains the qPCR analysis from CHOP expression in Figure 1—figure supplement 2B, and brightfield images of Trypan Blue staining measured on the Countess II for n = 3 biological replicates, summarized in Figure 1—figure supplement 2D. [file elife-52291-fig1-data6.zip › Figure 1 - Source Data 6/Source Data Fig 1S2D - Trypan Blue for CHOP expression/hct mpz.jpg]

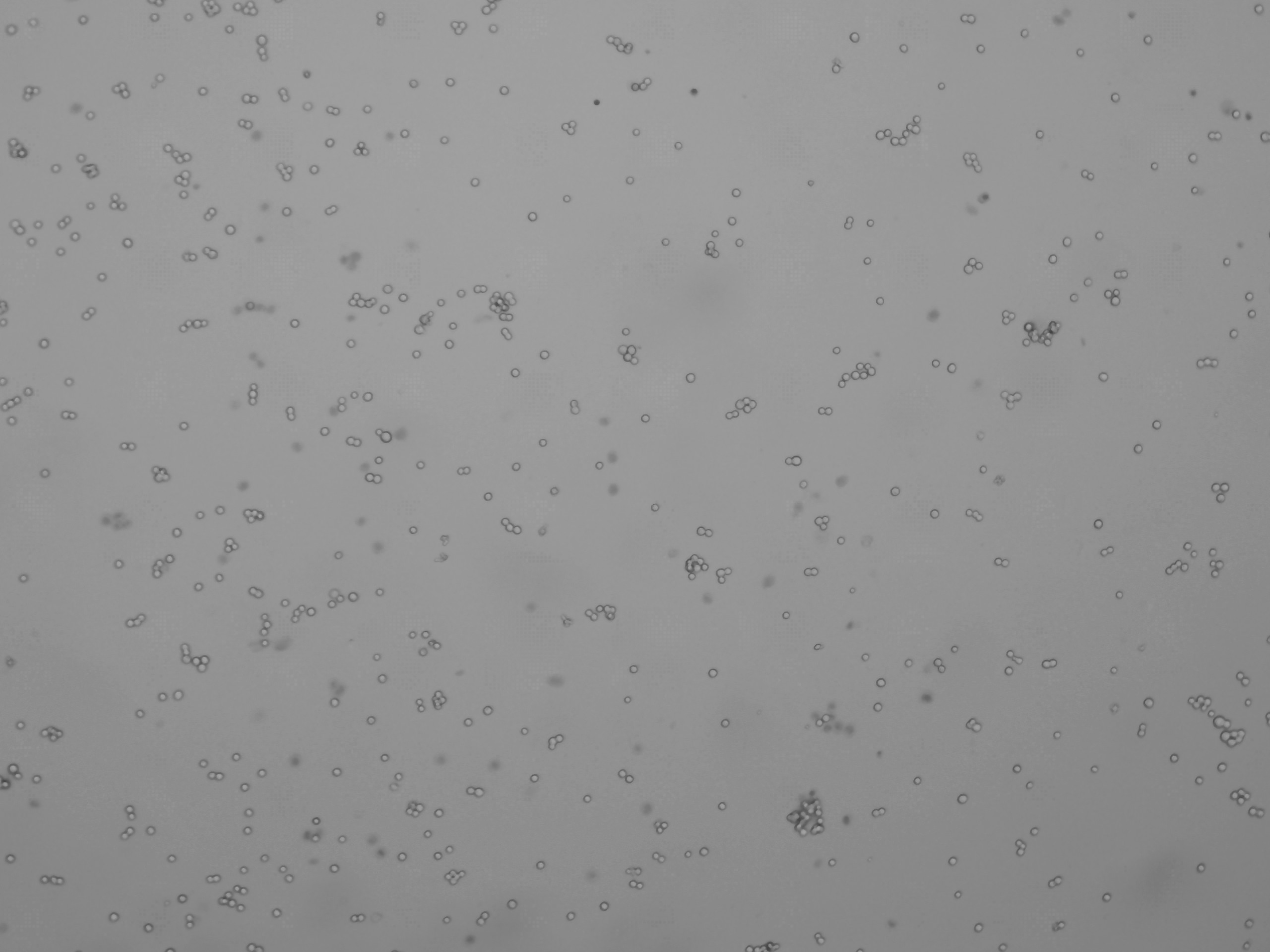

Supplement: Figure 1—source data 6. — This zip archive contains the qPCR analysis from CHOP expression in Figure 1—figure supplement 2B, and brightfield images of Trypan Blue staining measured on the Countess II for n = 3 biological replicates, summarized in Figure 1—figure supplement 2D. [file elife-52291-fig1-data6.zip › Figure 1 - Source Data 6/Source Data Fig 1S2D - Trypan Blue for CHOP expression/20190523 hct chop 250 rep 2_BF.jpg]

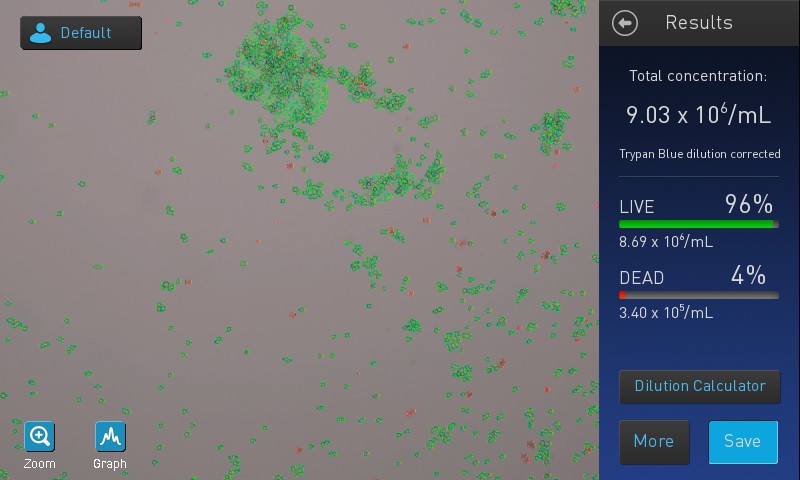

Supplement: Figure 1—source data 6. — This zip archive contains the qPCR analysis from CHOP expression in Figure 1—figure supplement 2B, and brightfield images of Trypan Blue staining measured on the Countess II for n = 3 biological replicates, summarized in Figure 1—figure supplement 2D. [file elife-52291-fig1-data6.zip › Figure 1 - Source Data 6/Source Data Fig 1S2D - Trypan Blue for CHOP expression/20190523 hct chop 125 rep 2.jpg]

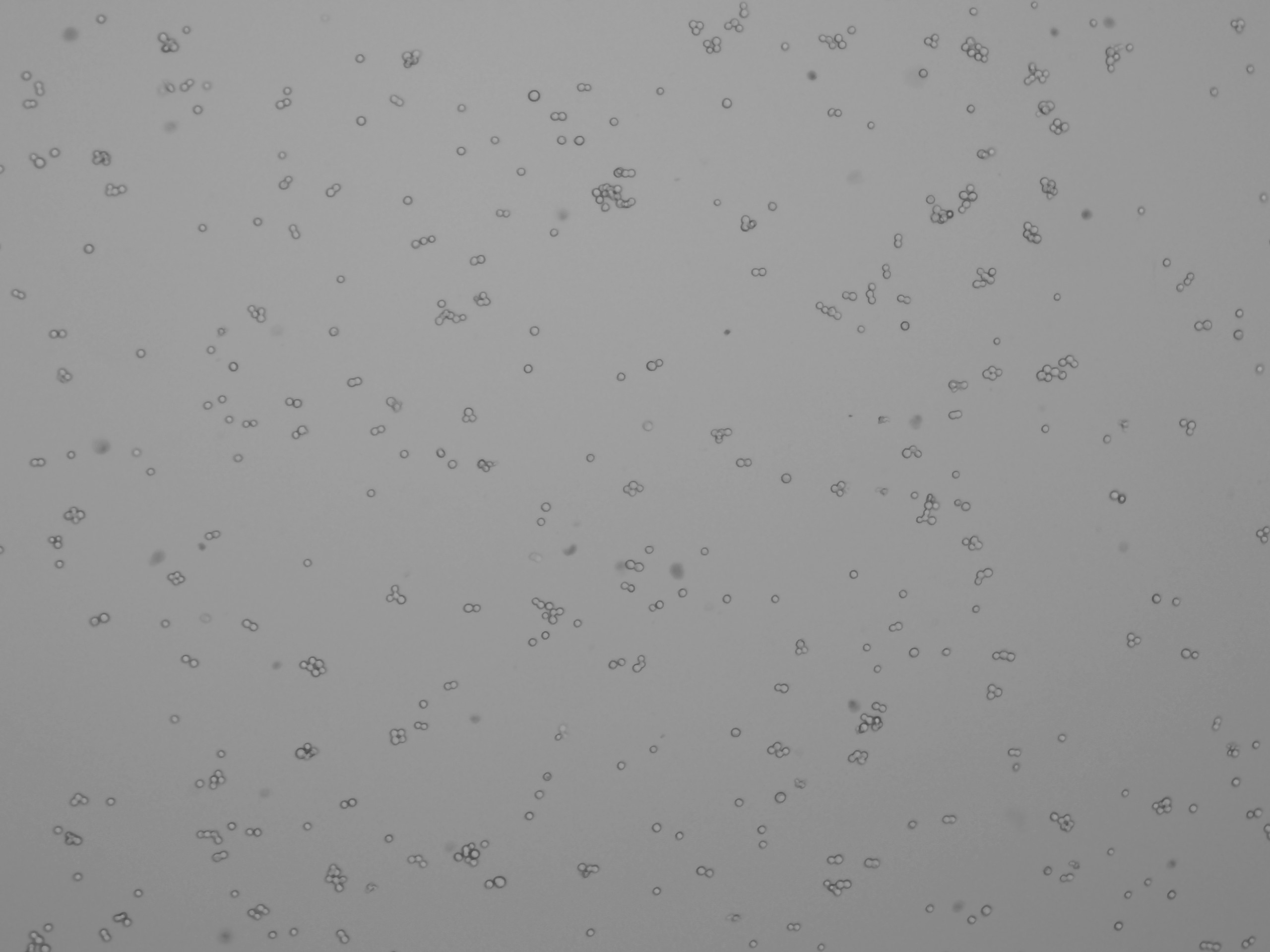

Supplement: Figure 1—source data 6. — This zip archive contains the qPCR analysis from CHOP expression in Figure 1—figure supplement 2B, and brightfield images of Trypan Blue staining measured on the Countess II for n = 3 biological replicates, summarized in Figure 1—figure supplement 2D. [file elife-52291-fig1-data6.zip › Figure 1 - Source Data 6/Source Data Fig 1S2D - Trypan Blue for CHOP expression/20190523 hct chop 1000 rep 3_BF.jpg]

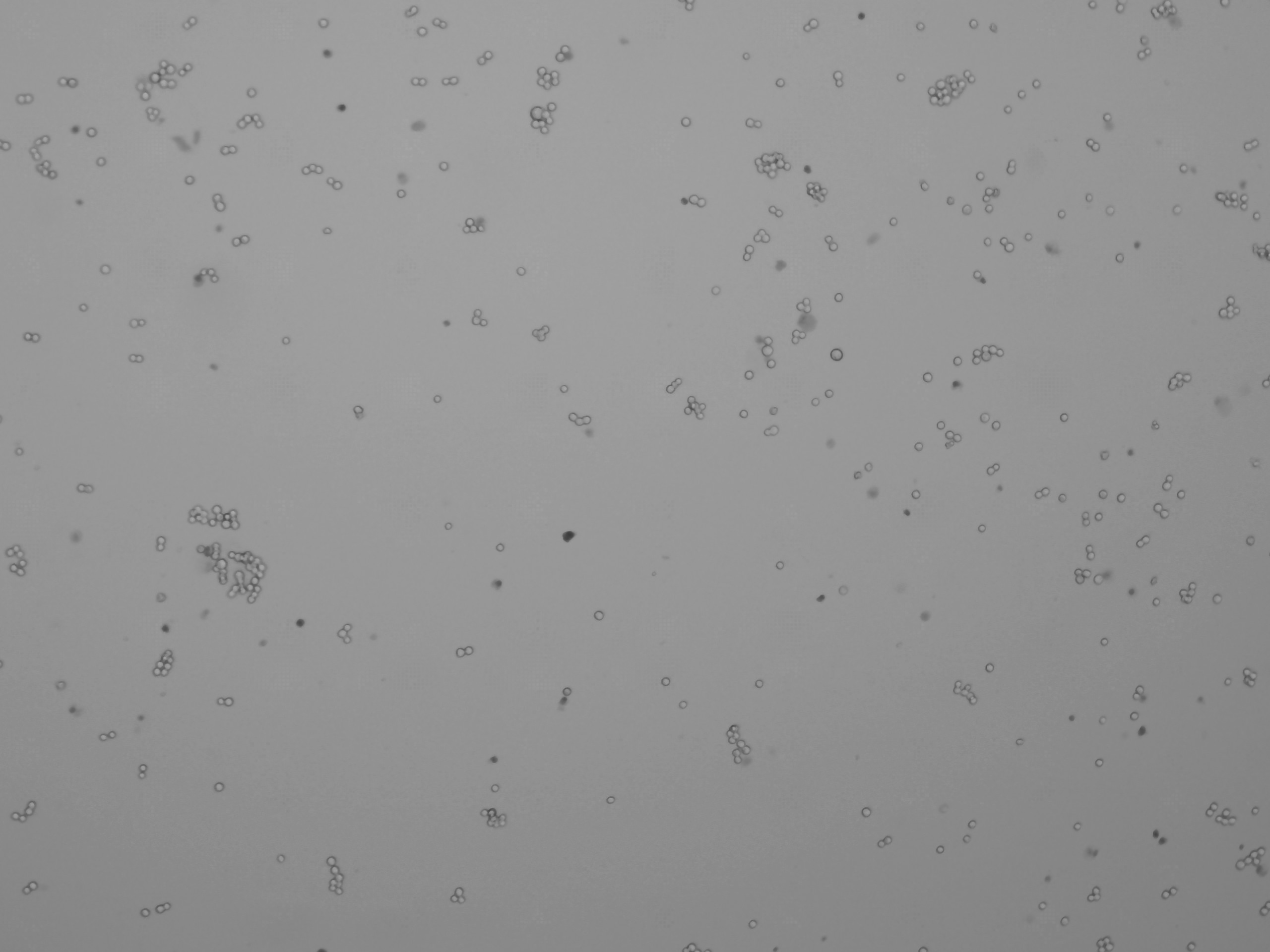

Supplement: Figure 1—source data 6. — This zip archive contains the qPCR analysis from CHOP expression in Figure 1—figure supplement 2B, and brightfield images of Trypan Blue staining measured on the Countess II for n = 3 biological replicates, summarized in Figure 1—figure supplement 2D. [file elife-52291-fig1-data6.zip › Figure 1 - Source Data 6/Source Data Fig 1S2D - Trypan Blue for CHOP expression/hct chop 250_BF.jpg]

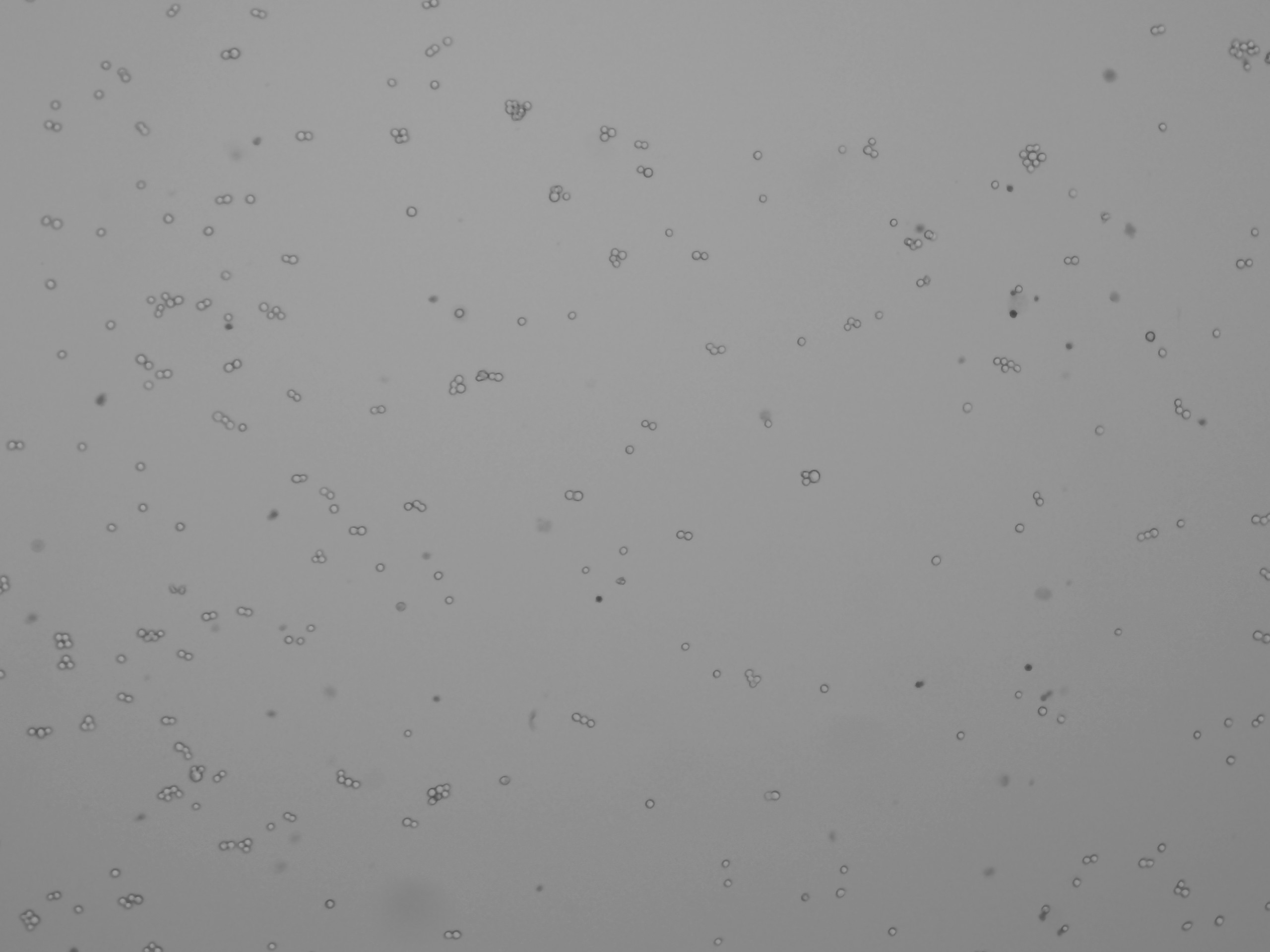

Supplement: Figure 1—source data 6. — This zip archive contains the qPCR analysis from CHOP expression in Figure 1—figure supplement 2B, and brightfield images of Trypan Blue staining measured on the Countess II for n = 3 biological replicates, summarized in Figure 1—figure supplement 2D. [file elife-52291-fig1-data6.zip › Figure 1 - Source Data 6/Source Data Fig 1S2D - Trypan Blue for CHOP expression/hct chop 62_BF.jpg]

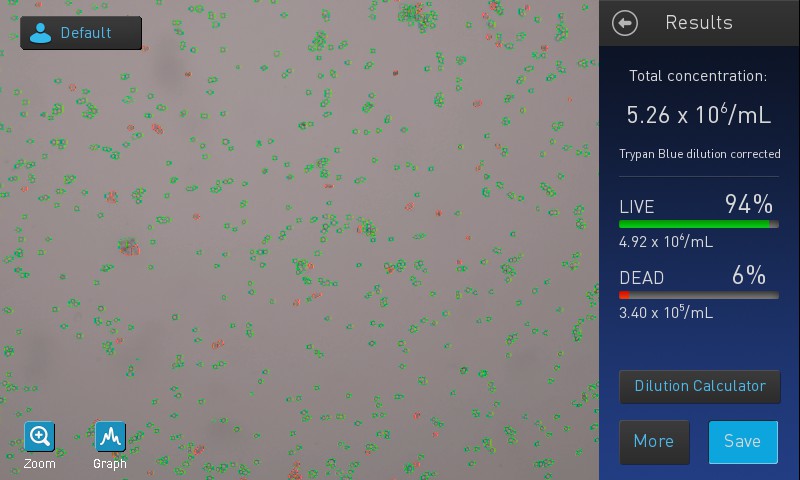

Supplement: Figure 1—source data 6. — This zip archive contains the qPCR analysis from CHOP expression in Figure 1—figure supplement 2B, and brightfield images of Trypan Blue staining measured on the Countess II for n = 3 biological replicates, summarized in Figure 1—figure supplement 2D. [file elife-52291-fig1-data6.zip › Figure 1 - Source Data 6/Source Data Fig 1S2D - Trypan Blue for CHOP expression/20190523 hct chop 500 rep 3.jpg]

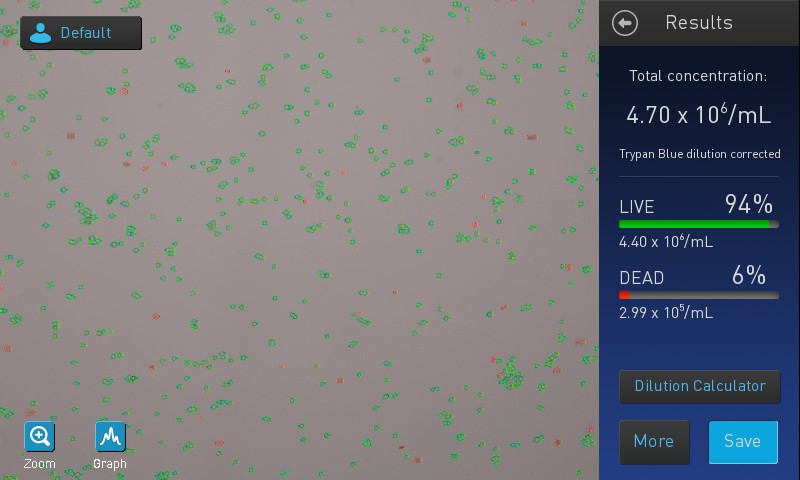

Supplement: Figure 1—source data 6. — This zip archive contains the qPCR analysis from CHOP expression in Figure 1—figure supplement 2B, and brightfield images of Trypan Blue staining measured on the Countess II for n = 3 biological replicates, summarized in Figure 1—figure supplement 2D. [file elife-52291-fig1-data6.zip › Figure 1 - Source Data 6/Source Data Fig 1S2D - Trypan Blue for CHOP expression/20190523 hct chop 500 rep 2.jpg]

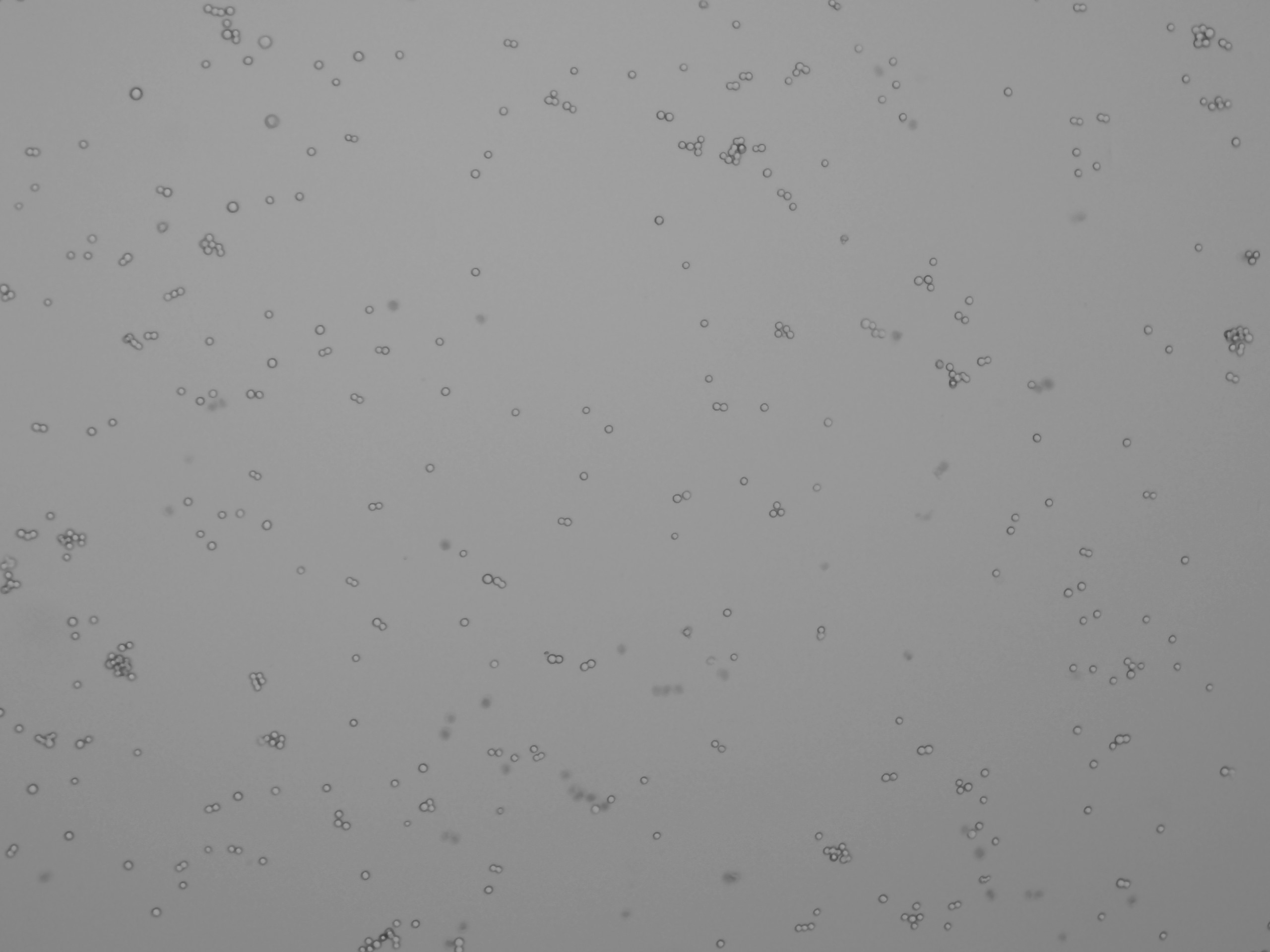

Supplement: Figure 1—source data 6. — This zip archive contains the qPCR analysis from CHOP expression in Figure 1—figure supplement 2B, and brightfield images of Trypan Blue staining measured on the Countess II for n = 3 biological replicates, summarized in Figure 1—figure supplement 2D. [file elife-52291-fig1-data6.zip › Figure 1 - Source Data 6/Source Data Fig 1S2D - Trypan Blue for CHOP expression/20190523 hct chop empty rep 3_BF.jpg]

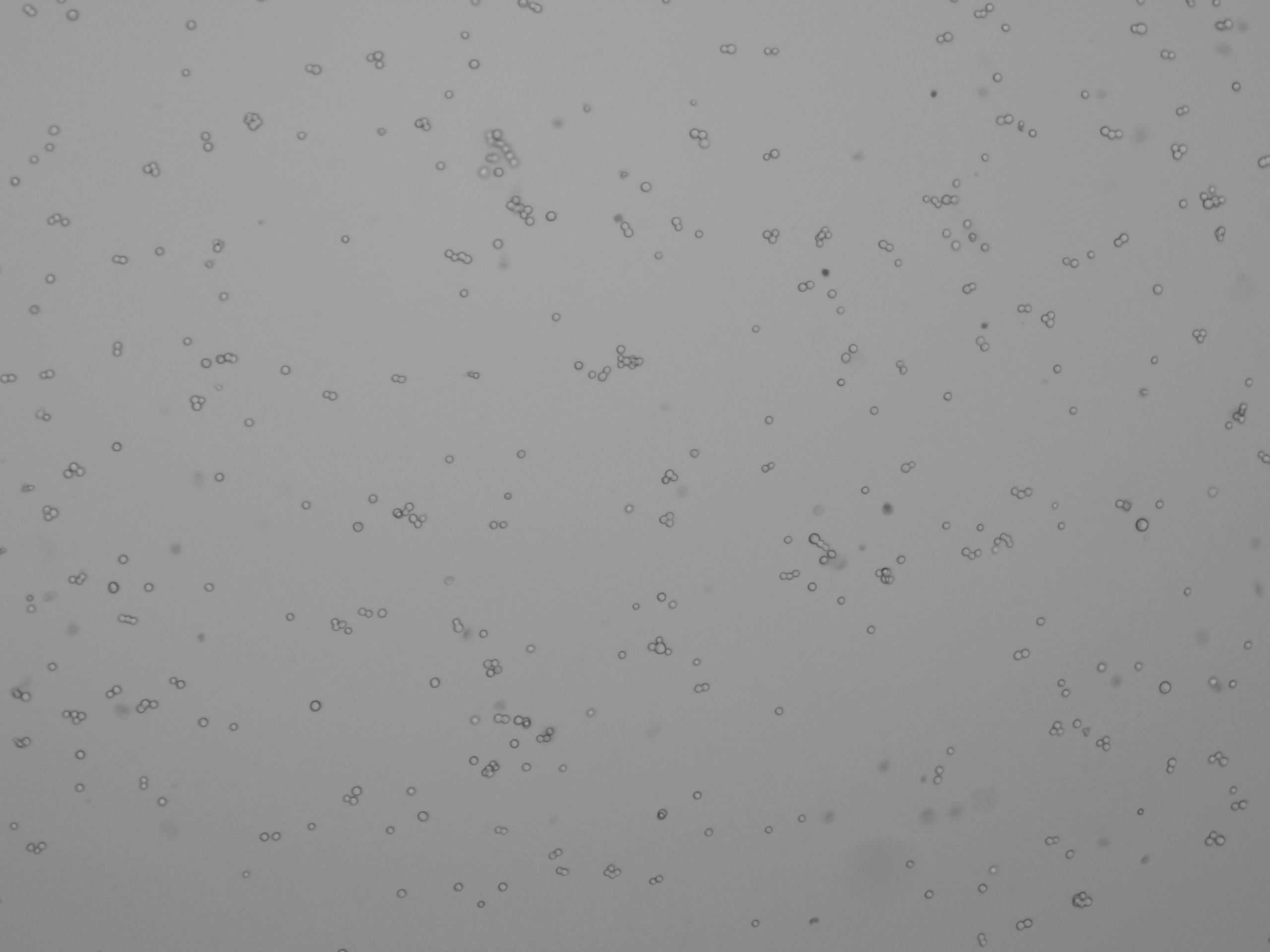

Supplement: Figure 1—source data 6. — This zip archive contains the qPCR analysis from CHOP expression in Figure 1—figure supplement 2B, and brightfield images of Trypan Blue staining measured on the Countess II for n = 3 biological replicates, summarized in Figure 1—figure supplement 2D. [file elife-52291-fig1-data6.zip › Figure 1 - Source Data 6/Source Data Fig 1S2D - Trypan Blue for CHOP expression/20190523 hct chop 1000 rep 1_BF.jpg]

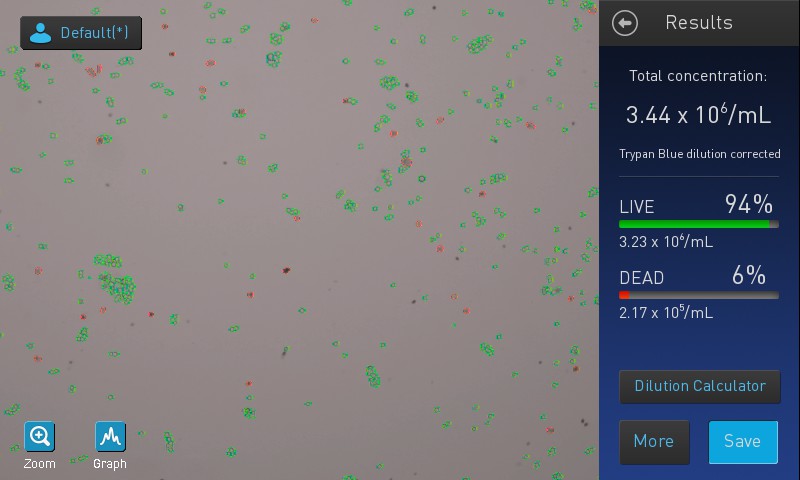

Supplement: Figure 1—source data 6. — This zip archive contains the qPCR analysis from CHOP expression in Figure 1—figure supplement 2B, and brightfield images of Trypan Blue staining measured on the Countess II for n = 3 biological replicates, summarized in Figure 1—figure supplement 2D. [file elife-52291-fig1-data6.zip › Figure 1 - Source Data 6/Source Data Fig 1S2D - Trypan Blue for CHOP expression/hct chop 250.jpg]

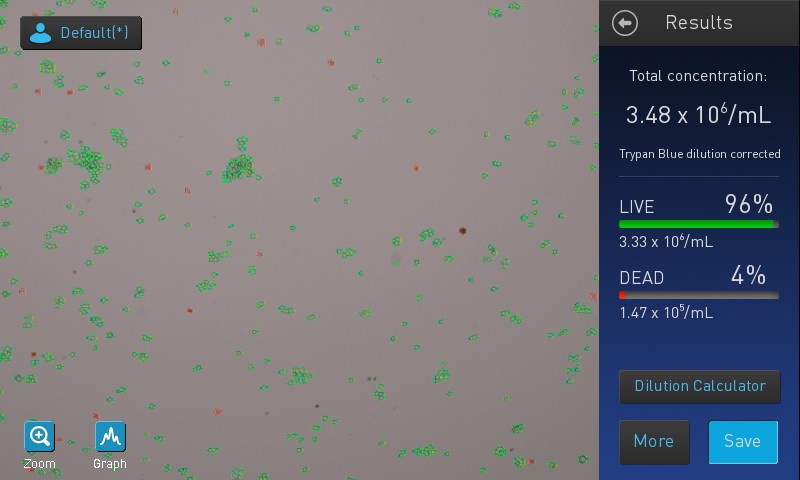

Supplement: Figure 1—source data 6. — This zip archive contains the qPCR analysis from CHOP expression in Figure 1—figure supplement 2B, and brightfield images of Trypan Blue staining measured on the Countess II for n = 3 biological replicates, summarized in Figure 1—figure supplement 2D. [file elife-52291-fig1-data6.zip › Figure 1 - Source Data 6/Source Data Fig 1S2D - Trypan Blue for CHOP expression/hct chop 125.jpg]

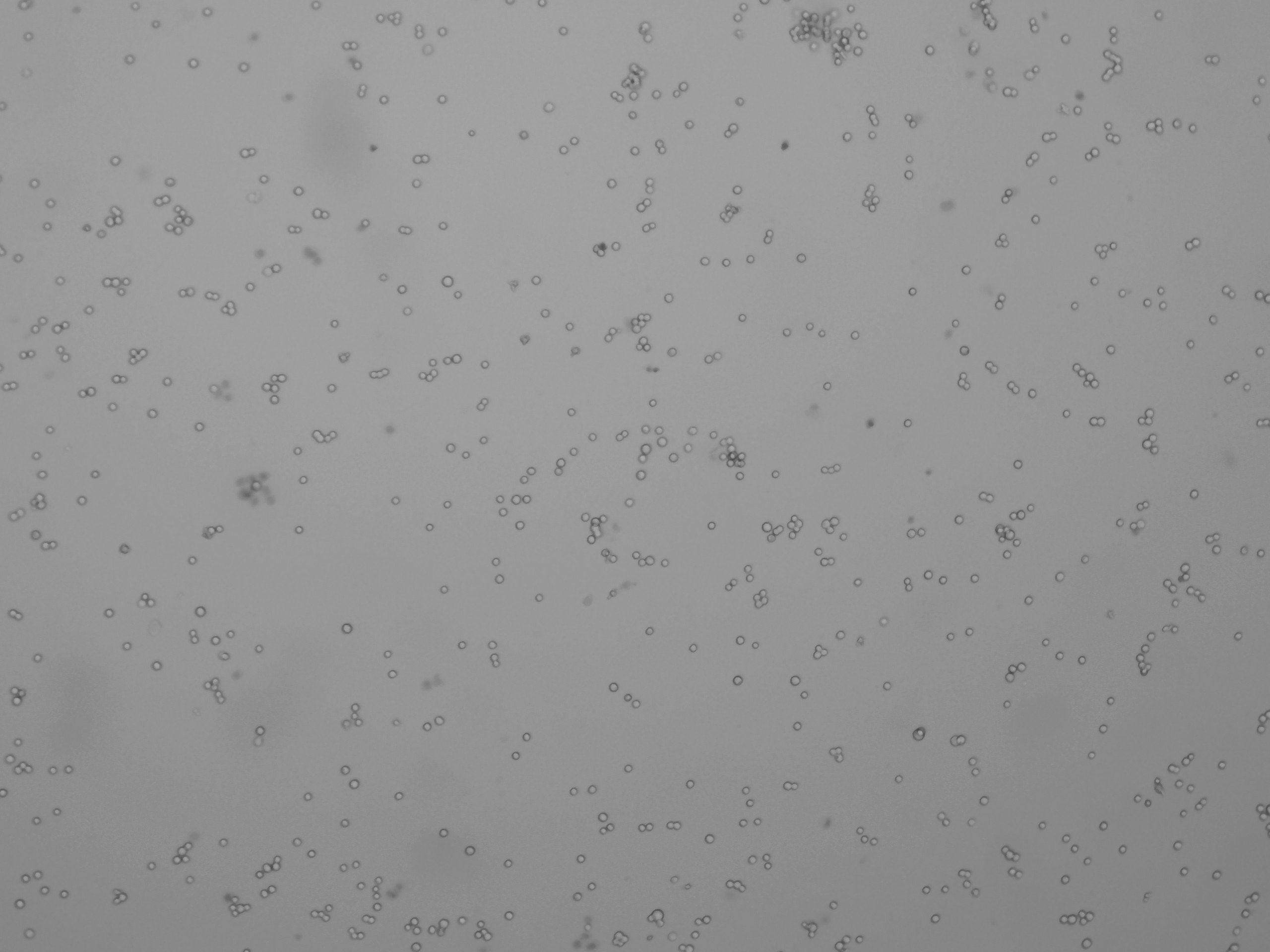

Supplement: Figure 1—source data 6. — This zip archive contains the qPCR analysis from CHOP expression in Figure 1—figure supplement 2B, and brightfield images of Trypan Blue staining measured on the Countess II for n = 3 biological replicates, summarized in Figure 1—figure supplement 2D. [file elife-52291-fig1-data6.zip › Figure 1 - Source Data 6/Source Data Fig 1S2D - Trypan Blue for CHOP expression/20190523 hct chop 500 rep 3_BF.jpg]

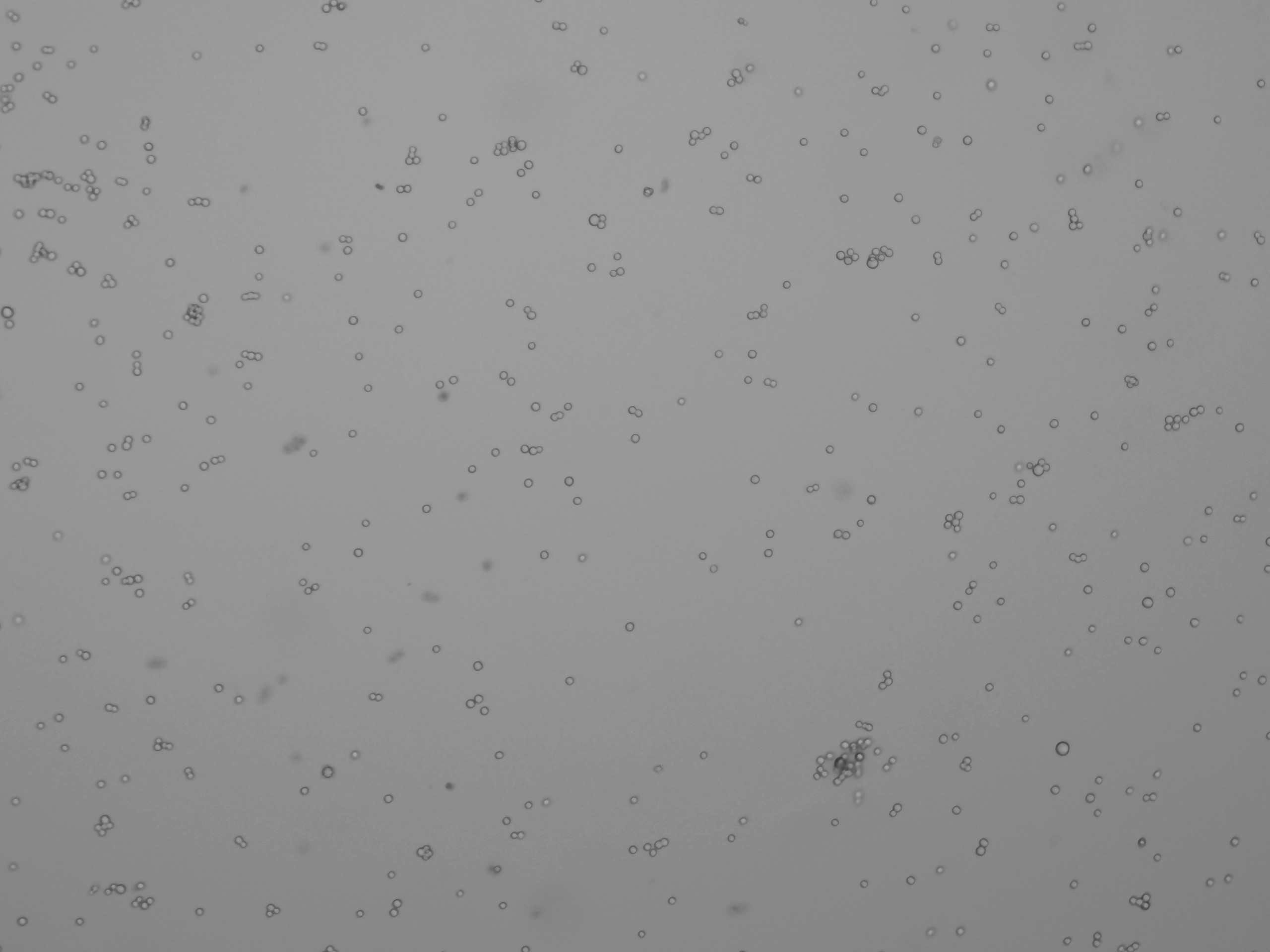

Supplement: Figure 1—source data 6. — This zip archive contains the qPCR analysis from CHOP expression in Figure 1—figure supplement 2B, and brightfield images of Trypan Blue staining measured on the Countess II for n = 3 biological replicates, summarized in Figure 1—figure supplement 2D. [file elife-52291-fig1-data6.zip › Figure 1 - Source Data 6/Source Data Fig 1S2D - Trypan Blue for CHOP expression/20190523 hct empty rep 2_BF.jpg]

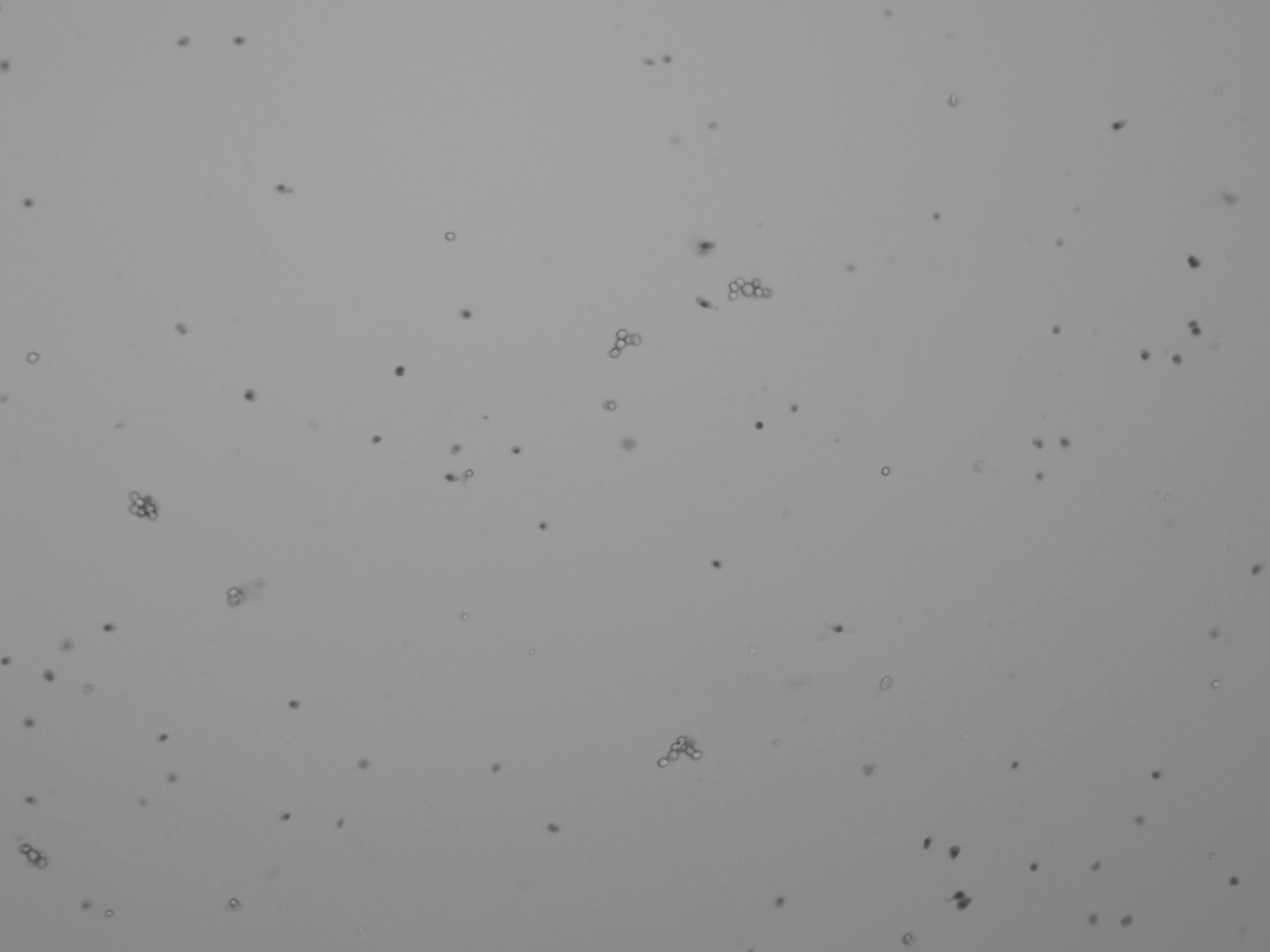

Supplement: Figure 1—source data 6. — This zip archive contains the qPCR analysis from CHOP expression in Figure 1—figure supplement 2B, and brightfield images of Trypan Blue staining measured on the Countess II for n = 3 biological replicates, summarized in Figure 1—figure supplement 2D. [file elife-52291-fig1-data6.zip › Figure 1 - Source Data 6/Source Data Fig 1S2D - Trypan Blue for CHOP expression/hct mpz_BF.jpg]

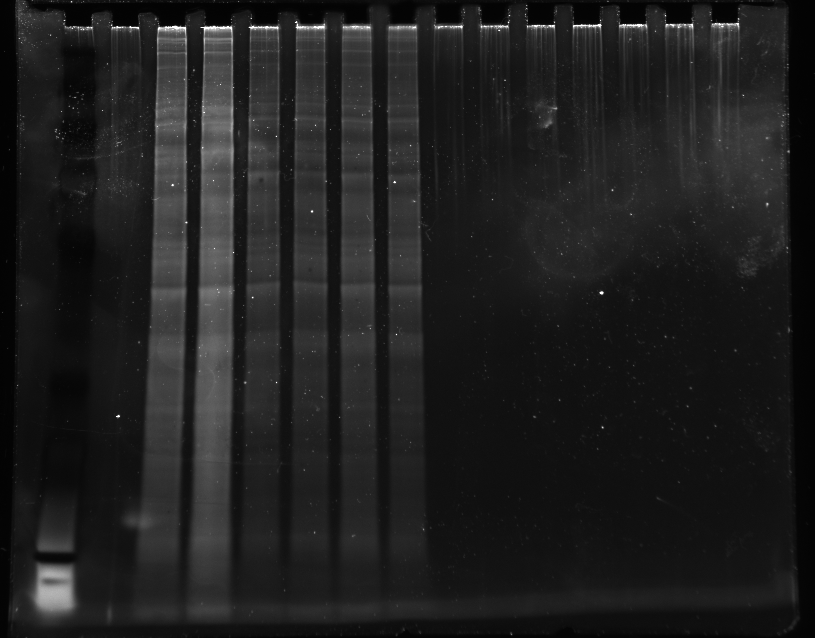

Supplement: Figure 1—source data 9. — This zip archive contains the measured luminescent units for caspase glo 8 activity shown in Figures 1S5B (input lysates and IP beads). Coomassie gels used to normalize lysate concentration are included as. tif files. [file elife-52291-fig1-data9.zip › Figure 1 - Source Data 9/Coomassie gels for lysates/2018-08-14_SyproRuby_HCT_RHO_INS_Caspase_glo_8_lysate_quant.tif]

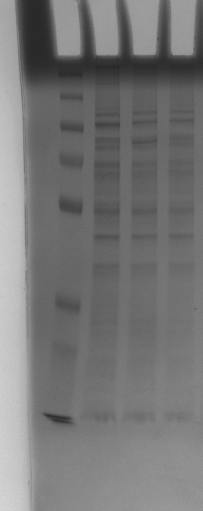

Supplement: Figure 1—source data 9. — This zip archive contains the measured luminescent units for caspase glo 8 activity shown in Figures 1S5B (input lysates and IP beads). Coomassie gels used to normalize lysate concentration are included as. tif files. [file elife-52291-fig1-data9.zip › Figure 1 - Source Data 9/Coomassie gels for lysates/2018-09-07 Coomassie for Lysates of HCT GFP INS RHO.tif]

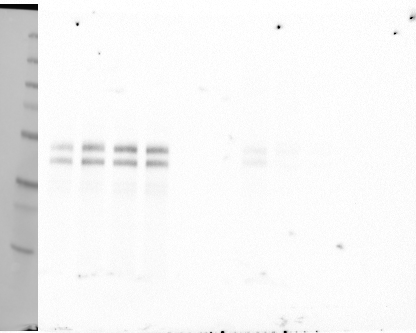

Supplement: Figure 4—source data 1. — This zip archive contains tif files of the Westerns from inputs and IPs of the MPZ-ecto peptides (n = 2 biological replicates) used to quantify the percent of DR5 recovered shown in Figure 4—figure supplement 3A. [file elife-52291-fig4-data1.zip › Figure 4 - Source Data 1/HCT116 ecto peptide IP Rep 2/antiDR5 HCT116 ecto peptide-GFP IP_Exposure_100.0sec-Rep 2.tif]

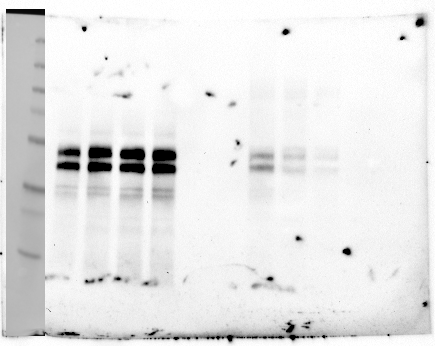

Supplement: Figure 4—source data 1. — This zip archive contains tif files of the Westerns from inputs and IPs of the MPZ-ecto peptides (n = 2 biological replicates) used to quantify the percent of DR5 recovered shown in Figure 4—figure supplement 3A. [file elife-52291-fig4-data1.zip › Figure 4 - Source Data 1/HCT116 ecto peptide IP Rep 2/antiDR5 HCT116 ecto peptide-GFP IP_Exposure_1000.0sec-Rep 2.tif]

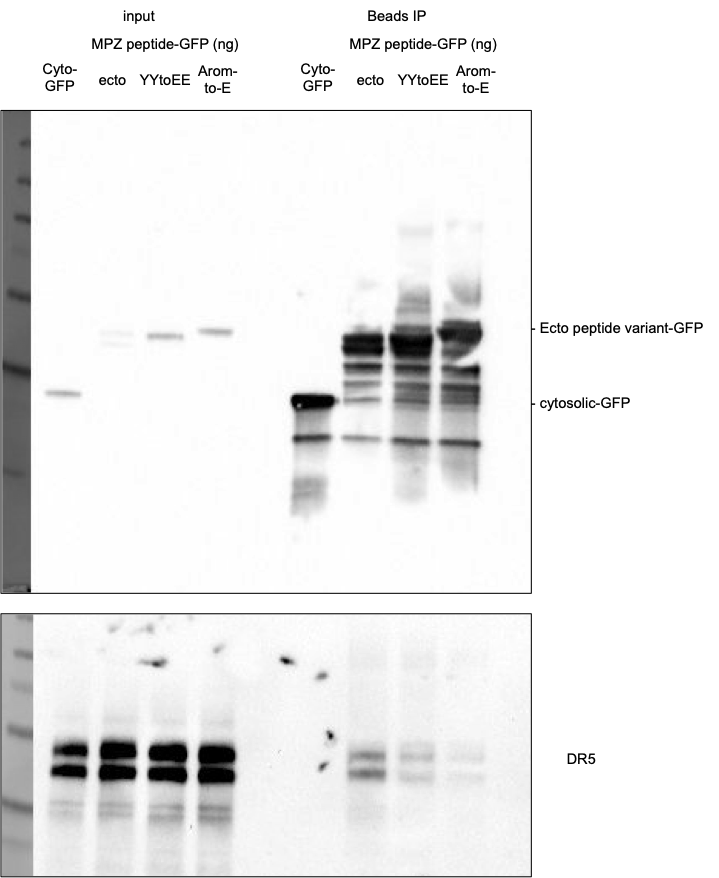

Supplement: Figure 4—source data 1. — This zip archive contains tif files of the Westerns from inputs and IPs of the MPZ-ecto peptides (n = 2 biological replicates) used to quantify the percent of DR5 recovered shown in Figure 4—figure supplement 3A. [file elife-52291-fig4-data1.zip › Figure 4 - Source Data 1/HCT116 ecto peptide IP Rep 2/Legend - HCT116 ecto peptide GFP IP Rep 2.png]

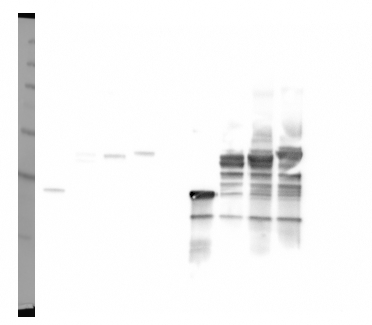

Supplement: Figure 4—source data 1. — This zip archive contains tif files of the Westerns from inputs and IPs of the MPZ-ecto peptides (n = 2 biological replicates) used to quantify the percent of DR5 recovered shown in Figure 4—figure supplement 3A. [file elife-52291-fig4-data1.zip › Figure 4 - Source Data 1/HCT116 ecto peptide IP Rep 2/antiGFP HCT116 ecto peptide GFP IP_Exposure_10.0sec-Rep 2.tif]

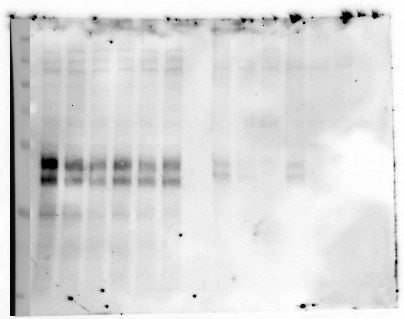

Supplement: Figure 4—source data 1. — This zip archive contains tif files of the Westerns from inputs and IPs of the MPZ-ecto peptides (n = 2 biological replicates) used to quantify the percent of DR5 recovered shown in Figure 4—figure supplement 3A. [file elife-52291-fig4-data1.zip › Figure 4 - Source Data 1/HCT116 ecto peptide IP Rep 1/anti-DR5 GFP IP HCT ecto peptide variants_Exposure_400.0sec-Rep 1.tif]

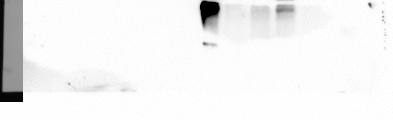

Supplement: Figure 4—source data 1. — This zip archive contains tif files of the Westerns from inputs and IPs of the MPZ-ecto peptides (n = 2 biological replicates) used to quantify the percent of DR5 recovered shown in Figure 4—figure supplement 3A. [file elife-52291-fig4-data1.zip › Figure 4 - Source Data 1/HCT116 ecto peptide IP Rep 1/antiFADD GFP IP HCT ecto peptide variants_Exposure_100.0sec-Rep 1.tif]

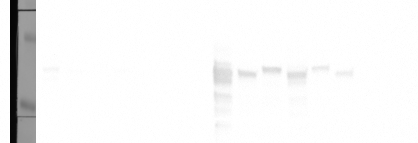

Supplement: Figure 4—source data 1. — This zip archive contains tif files of the Westerns from inputs and IPs of the MPZ-ecto peptides (n = 2 biological replicates) used to quantify the percent of DR5 recovered shown in Figure 4—figure supplement 3A. [file elife-52291-fig4-data1.zip › Figure 4 - Source Data 1/HCT116 ecto peptide IP Rep 1/antiGFP GFP IP HCT ecto peptide variants_Exposure_18.0sec-Rep 1.tif]

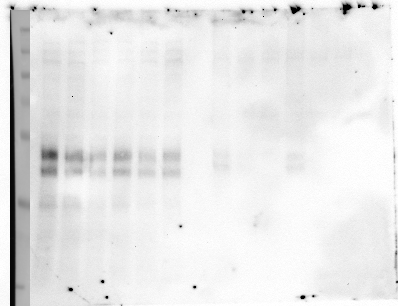

Supplement: Figure 4—source data 1. — This zip archive contains tif files of the Westerns from inputs and IPs of the MPZ-ecto peptides (n = 2 biological replicates) used to quantify the percent of DR5 recovered shown in Figure 4—figure supplement 3A. [file elife-52291-fig4-data1.zip › Figure 4 - Source Data 1/HCT116 ecto peptide IP Rep 1/anti-DR5 GFP IP HCT ecto peptide variants_Exposure_200.0sec-Rep 1.tif]

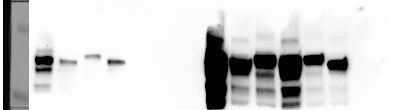

Supplement: Figure 4—source data 1. — This zip archive contains tif files of the Westerns from inputs and IPs of the MPZ-ecto peptides (n = 2 biological replicates) used to quantify the percent of DR5 recovered shown in Figure 4—figure supplement 3A. [file elife-52291-fig4-data1.zip › Figure 4 - Source Data 1/HCT116 ecto peptide IP Rep 1/antiGFP GFP IP HCT ecto peptide variants_Exposure_100.0sec-Rep 1.tif]

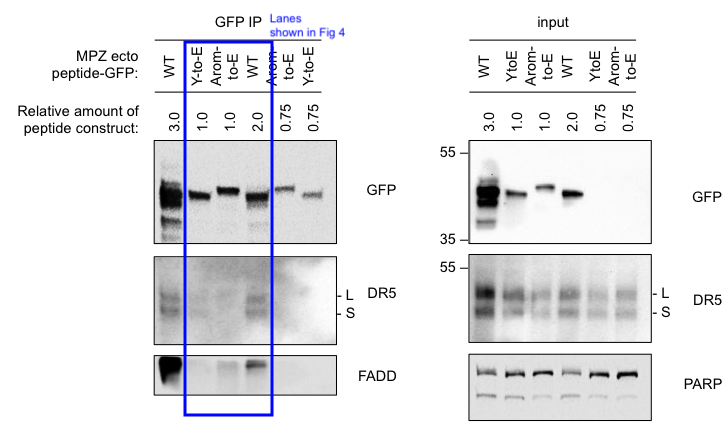

Supplement: Figure 4—source data 1. — This zip archive contains tif files of the Westerns from inputs and IPs of the MPZ-ecto peptides (n = 2 biological replicates) used to quantify the percent of DR5 recovered shown in Figure 4—figure supplement 3A. [file elife-52291-fig4-data1.zip › Figure 4 - Source Data 1/HCT116 ecto peptide IP Rep 1/Legend - HCT116 ecto peptide GFP IP Rep 1.png]

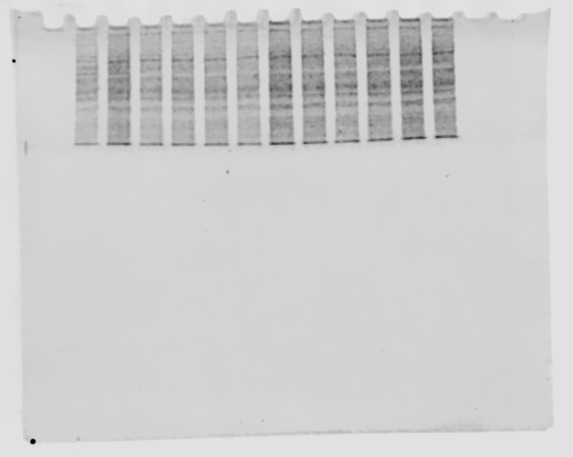

Supplement: Figure 4—source data 2. — This zip archive contains the measured luminescent units for caspase glo 8 activity shown in Figure 4C (lysates) and the coomassie gel used to normalize lysate concentration as a.tif file. [file elife-52291-fig4-data2.zip › Figure 4 - Source Data 2/Source Data Fig 4C - Coomassie Rep 1.tif]

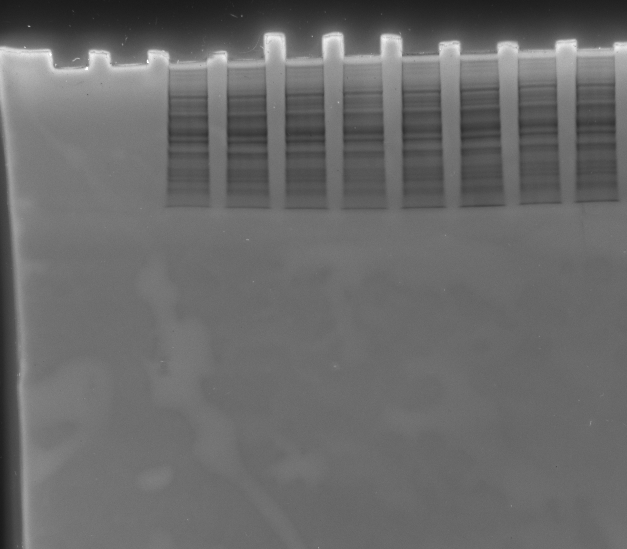

Supplement: Figure 4—source data 2. — This zip archive contains the measured luminescent units for caspase glo 8 activity shown in Figure 4C (lysates) and the coomassie gel used to normalize lysate concentration as a.tif file. [file elife-52291-fig4-data2.zip › Figure 4 - Source Data 2/Source Data Fig 4C - Coomassie Rep 2.tif]
