# Supplementary material for: Misfolded proteins bind and activate death receptor 5 to trigger apoptosis during unresolved endoplasmic reticulum stress
Source: eLife. 2020 Jan 6;9:e52291. doi: 10.7554/eLife.52291 (PMC7041945; doi:10.7554/eLife.52291)
Supplement: Figure 1—source data 6. — This zip archive contains the qPCR analysis from CHOP expression in Figure 1—figure supplement 2B, and brightfield images of Trypan Blue staining measured on the Countess II for n = 3 biological replicates, summarized in Figure 1—figure supplement 2D. [file elife-52291-fig1-data6.zip › Figure 1 - Source Data 6/Source Data Fig 1S2D - Trypan Blue for CHOP expression/20190523 hct chop 250 rep 2_R.pdf]

# Countess II Live/Dead Report

File name: 20190523 hct chop 250 rep 2_R.pdf

Date: 05.24.2019 08:22:44 AM

## Results:

| | | Concentration |
|---|---|---|
| Total | | $4.73 \times 10^6$/mL |
| Live | 88% | $4.15 \times 10^6$/mL |
| Dead | 12% | $5.81 \times 10^5$/mL |

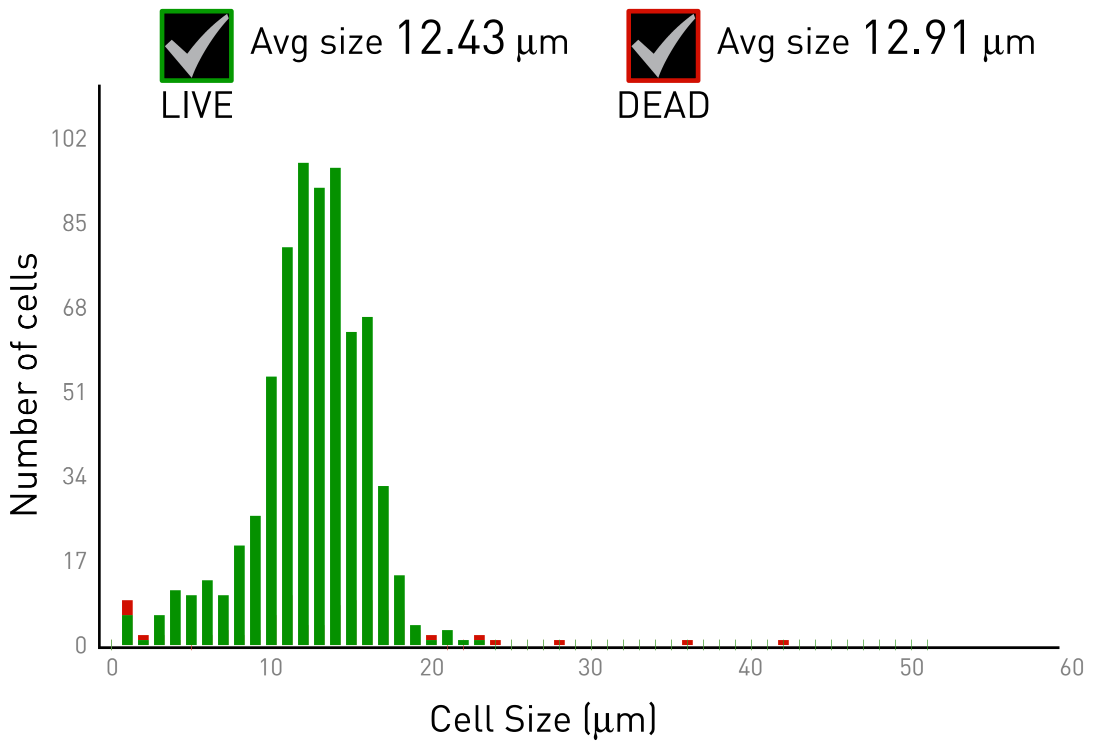


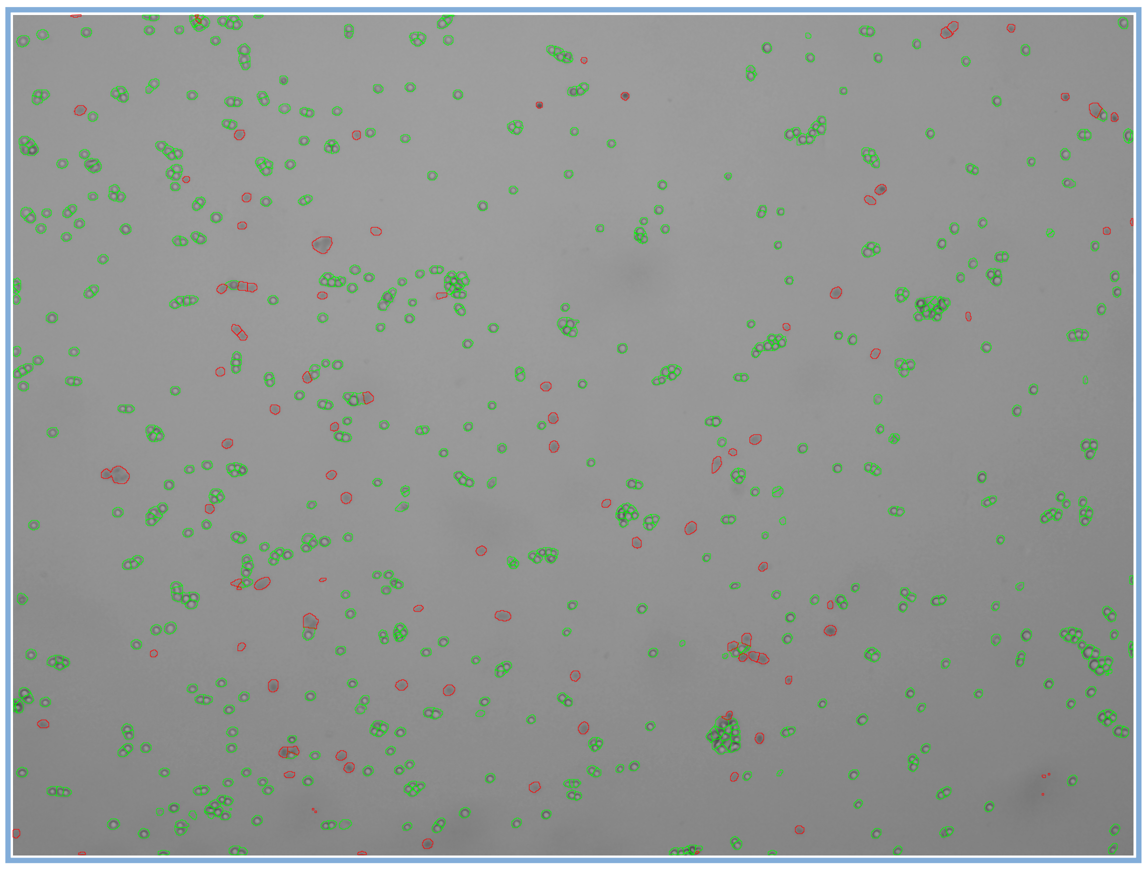

## Profile: Default

| | | Live | Dead |
|---|---|---|---|
| Auto FL threshold: On | | | |
| Auto lighting: On | Size: | 0 , 60 | 0 , 60 |
| | Brightness: | 0 , 255 | 0 , 255 |
| | Circularity: | 0.47 | 0.57 |
