# Supplementary material for: Misfolded proteins bind and activate death receptor 5 to trigger apoptosis during unresolved endoplasmic reticulum stress
Source: eLife. 2020 Jan 6;9:e52291. doi: 10.7554/eLife.52291 (PMC7041945; doi:10.7554/eLife.52291)
Supplement: Figure 1—source data 6. — This zip archive contains the qPCR analysis from CHOP expression in Figure 1—figure supplement 2B, and brightfield images of Trypan Blue staining measured on the Countess II for n = 3 biological replicates, summarized in Figure 1—figure supplement 2D. [file elife-52291-fig1-data6.zip › Figure 1 - Source Data 6/Source Data Fig 1S2D - Trypan Blue for CHOP expression/20190523 hct chop 1000 rep 3_R.pdf]

Countess II Live/Dead Report

File name: 20190523 hct chop 1000 rep 3_R.pdf

Date: 05.24.2019 08:19:30 AM

Results:

		Concentration
Total		4.14×10^6/mL
Live	95%	3.95×10^6/mL
Dead	5%	1.88×10^5/mL

LIVE Avg size 11.65 µm

DEAD Avg size 13.12 µm

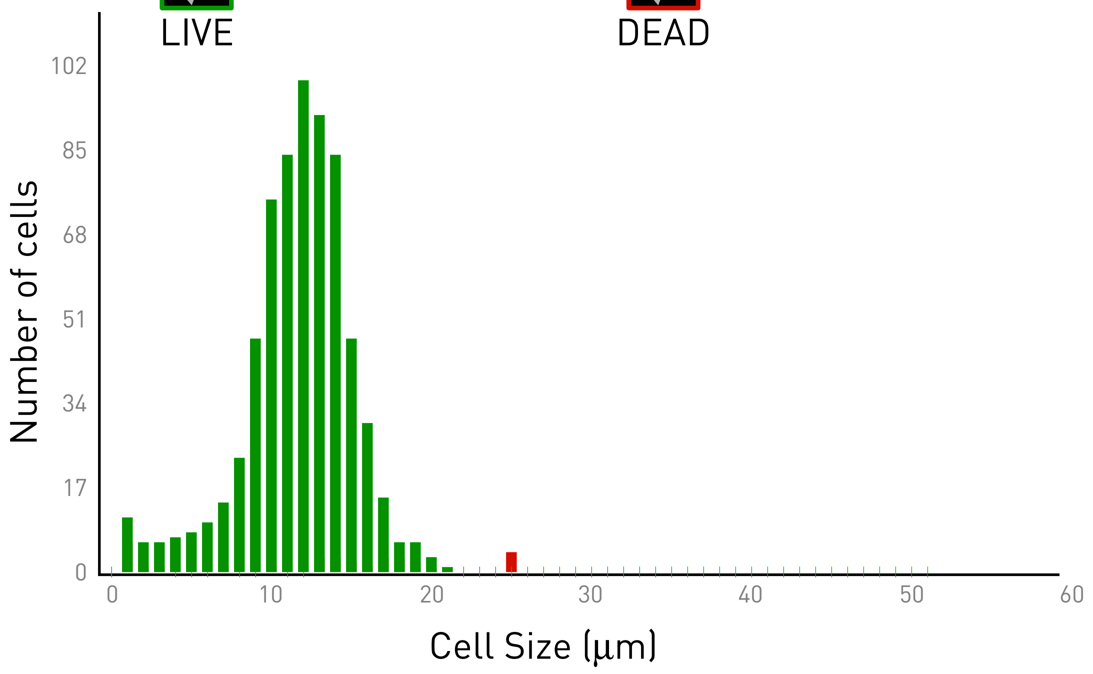

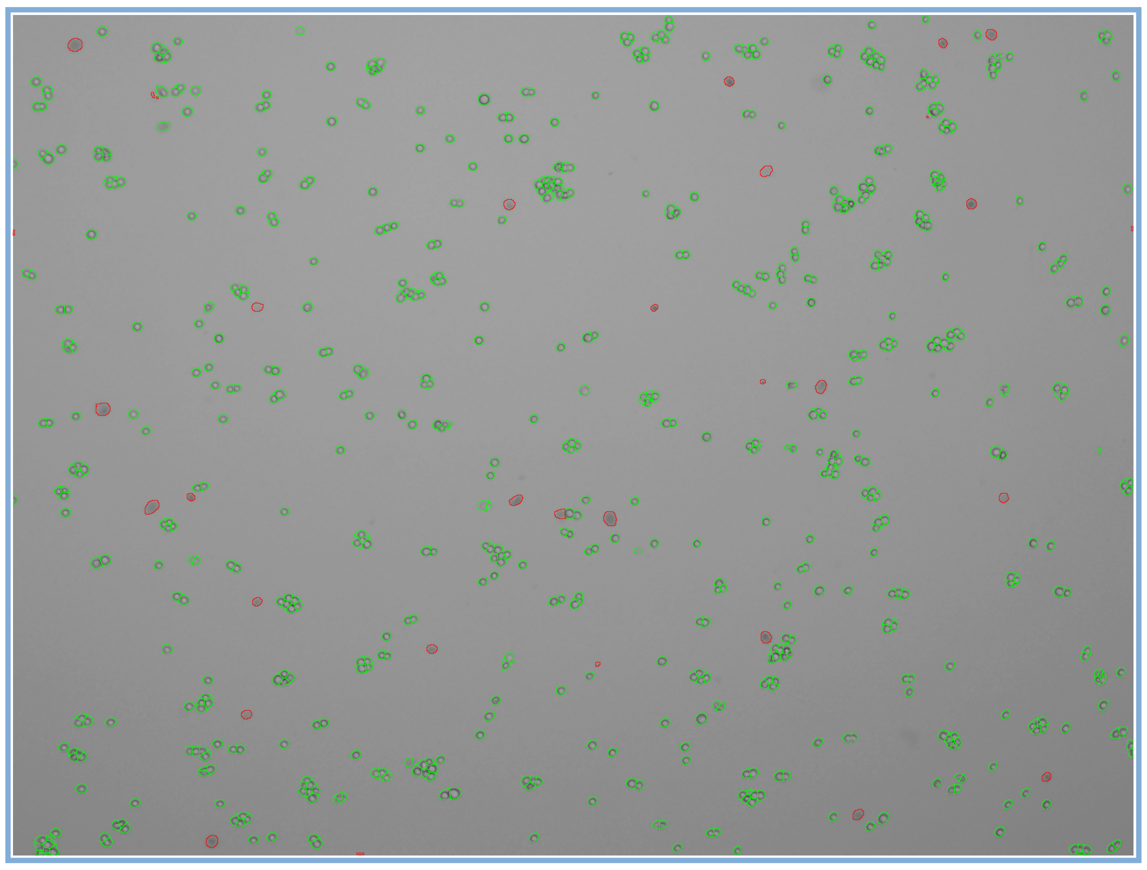

Profile: Default

		Live	Dead
Auto FL threshold: On			
Auto lighting: On	Size:	0 , 60	0 , 60
	Brightness:	0 , 255	0 , 255
	Circularity:	0.41	0.49
